# Structural Basis for Type VI Secretion Effector Recognition by a Cognate Immunity Protein

**DOI:** 10.1371/journal.ppat.1002613

**Published:** 2012-04-12

**Authors:** Mo Li, Isolde Le Trong, Mike A. Carl, Eric T. Larson, Seemay Chou, Justin A. De Leon, Simon L. Dove, Ronald E. Stenkamp, Joseph D. Mougous

**Affiliations:** 1 Department of Microbiology, University of Washington, Seattle, Washington, United States of America; 2 Department of Biological Structure, University of Washington, Seattle, Washington, United States of America; 3 Department of Biochemistry, University of Washington, Seattle, Washington, United States of America; 4 Division of Infectious Diseases, Children's Hospital, Harvard Medical School, Boston, Massachusetts, United States of America; Yale University School of Medicine, United States of America

## Abstract

The type VI secretion system (T6SS) has emerged as an important mediator of interbacterial interactions. A T6SS from *Pseudomonas aeruginosa* targets at least three effector proteins, type VI secretion exported 1–3 (Tse1–3), to recipient Gram-negative cells. The Tse2 protein is a cytoplasmic effector that acts as a potent inhibitor of target cell proliferation, thus providing a pronounced fitness advantage for *P. aeruginosa* donor cells. *P. aeruginosa* utilizes a dedicated immunity protein, type VI secretion immunity 2 (Tsi2), to protect against endogenous and intercellularly-transferred Tse2. Here we show that Tse2 delivered by the T6SS efficiently induces quiescence, not death, within recipient cells. We demonstrate that despite direct interaction of Tsi2 and Tse2 in the cytoplasm, Tsi2 is dispensable for targeting the toxin to the secretory apparatus. To gain insights into the molecular basis of Tse2 immunity, we solved the 1.00 Å X-ray crystal structure of Tsi2. The structure shows that Tsi2 assembles as a dimer that does not resemble previously characterized immunity or antitoxin proteins. A genetic screen for Tsi2 mutants deficient in Tse2 interaction revealed an acidic patch distal to the Tsi2 homodimer interface that mediates toxin interaction and immunity. Consistent with this finding, we observed that destabilization of the Tsi2 dimer does not impact Tse2 interaction. The molecular insights into Tsi2 structure and function garnered from this study shed light on the mechanisms of T6 effector secretion, and indicate that the Tse2–Tsi2 effector–immunity pair has features distinguishing it from previously characterized toxin–immunity and toxin–antitoxin systems.

## Author Summary

Bacterial species have been at war with each other for over a billion years. During this period they have evolved many pathways for besting the competition; one of the most recent of these to be described is the type VI secretion system (T6SS). The T6SS of *Pseudomonas aeruginosa* is a complex machine that the bacterium uses to intoxicate neighboring cells. Among the toxins this system delivers is type VI secretion exported 2 (Tse2). In addition to acting on competing organisms, this toxin can act on *P. aeruginosa*; thus, the organism synthesizes a protein, type VI secretion immunity 2 (Tsi2), which neutralizes the toxin. In this paper we dissect the function and structure of Tsi2. We show that although Tsi2 interacts with and stabilizes Tse2 inside the bacterium, the toxin does not require the immunity protein to reach the secretion apparatus. Our structure of Tsi2 shows that the protein adopts a dimeric configuration; however, we find that its dimerization is not required for Tse2 interaction. Instead, our findings indicate that Tse2 interacts with an acidic surface of Tsi2 that is opposite the homodimer interface. Our results provide key molecular insights into the process of T6 toxin secretion and immunity.

## Introduction

The type VI secretion system (T6SS) is a multifaceted protein export pathway that is widely distributed in Gram-negative Proteobacteria [1], [2]. With a minimal functional requirement for the products of at least 13 genes, this secretion machine rivals the complexity of the more extensively characterized type III and IV systems [3], [4]. Among the conserved components of the T6SS are a AAA+ family ATPase, ClpV, two proteins with sequence similarity to the type IVB secretion proteins IcmF and DotU, TssM and TssL, and several proteins with sequence or structural similarity to non-filamentous phage proteins [5]. The latter group of proteins includes Haemolysin co-regulated protein (Hcp) and Valine glycine repeat protein G (VgrG), which bear structural similarity to the tail protein of lambda phage (gpV) and the puncturing device of T4 phage (gp27/gp5), respectively [6]–[8]. Hcp and VgrG proteins are exported in a co-dependent fashion from nearly all T6SSs characterized to date. In combination, these observations have led to a prominent structure-function model in which the T6S apparatus resembles outward facing phage on the bacterial cell surface [9].

Early investigations of the T6SS focused on its role in modulating bacterial-host cell interactions. These efforts yielded information about the genetic requirements for T6S function and provided evidence that a subset of T6SSs influence pathogenesis by specifically mediating bacterial interactions with eukaryotic cells [10]. In addition to mediating host cell interactions, the T6SS has been shown to regulate gene expression and contribute to biofilm formation [11], [12]. It is not currently understood how the system mediates such diverse phenomena, nor is it known in all cases whether the effects observed are a direct or indirect result of its function.

Recently it has become clear that the T6SS plays a key role in mediating interactions between bacterial cells [2]. This was first observed in *P. aeruginosa*, where the Hcp secretion island I-encoded T6SS (H1-T6SS) was shown to target an effector protein, Tse2 (type VI secretion exported 2), to other *P. aeruginosa* cells [13]. Recipient cells lacking a Tse2-specific immunity protein, Tsi2 (type VI secretion immunity 2), were found to be at a competitive disadvantage relative to donor cells possessing Tse2. Although the mechanism of action of Tse2 remains unknown, the fitness advantage bestowed by the protein requires a functional T6SS in the donor cell and close association of donor and recipient cells. The H1-T6SS exports at least two additional effector proteins, Tse1 and Tse3 [14]. These proteins are targeted by the T6SS to the periplasm of recipient cells where they degrade peptidoglycan and thereby provide a competitive fitness advantage for *P. aeruginosa* donor cells. *P. aeruginosa* protects its own cells from the action of these toxic proteins by synthesizing cognate periplasmic immunity proteins, Tsi1 and Tsi3.

Tsi2 differs from Tsi1 and Tsi3 in several respects. For instance, Tsi2 is an essential protein in *P. aeruginosa*, whereas Tsi1 and Tsi3 are dispensable for growth [14]. This reflects the differences in the subcellular sites of action of the associated cognate toxins. Because the T6S export mechanism avoids periplasmic intermediates, immunity proteins for periplasmically-targeted effectors (Tse1 and Tse3) are required only for resisting intercellular self-intoxication. Conversely, Tse2 appears to act in the cytoplasm; therefore, in addition to resisting Tse2 delivered to the cytoplasm via intercellular self-intoxication, Tsi2 is essential for protecting against endogenous cytoplasmic intermediates of Tse2. Owing to their localization in the same subcellular compartment, Tse2 and Tsi2 are able to complex prior to toxin export. In the case of Tse1–Tsi1 and Tse3–Tsi3, the physical separation of the toxins (cytoplasmic) from their immunity proteins (periplasm) prevents such interactions. This likely imparts a unique requirement for Tse2 export – it must be recognized by the secretory apparatus in the context of a protein complex. Since Tsi2 is not exported by H1-T6SS, it must also be dissociated prior to or during the secretion of Tse2. In this way, Tsi2 is analogous to specialized dedicated secretion chaperones involved in the export of effectors from other alternative secretion pathways such as the type III and IV systems [3], [15]. Secretion chaperones are known to function critically both in stabilizing cognate effectors prior to export and in targeting effectors to the secretion apparatus.

It is apparent that bacterial genomes possess an enormous diversity of toxin-immunity modules outside of T6S-associated Tse–Tsi pairs [16]–[18]. Perhaps the most abundant and thoroughly characterized of these are the toxin-antitoxin (TA) systems [19], [20]. Growing evidence supports a general role for TA systems in resistance to stress and persister cell formation [21]. Type II TA systems consist of a cytoplasmic toxin that is maintained–under favorable conditions–in an inactive state by direct binding to a specific cognate antitoxin protein. Upon activation of cellular stress-response pathways, the antitoxin, which is typically less stable than the toxin, is rapidly degraded by cellular proteases including Lon (Long Form Filament), allowing the toxin to act on its target(s). Toxins vary in their mechanism, however most act as either ribosome-dependent or -independent mRNAses [22], [23].

The properties of the Tse2–Tsi2 pair that make it unique among T6S effector–immunity proteins are the same as those that offer analogy to effector–chaperone and toxin–antitoxin systems. In this report, we sought to ascertain the degree of similarity between these systems by interrogating the structure and function of Tsi2. Our results define properties of Tse2–Tsi2 that are shared with both TA and effector-chaperone systems, however we find that the Tse2–Tsi2 system is altogether functionally, structurally, and mechanistically distinct.

## Results

### Tsi2 protects against Tse2-induced stasis

We have reported that *P. aeruginosa* donor cells capable of delivering Tse2 by an active H1-T6SS to *P. aeruginosa* recipient bacteria lacking *tsi2* have a pronounced competitive fitness advantage [13]. However, absolute colony forming units (CFU) of competing bacteria were not determined in these experiments, which precluded defining whether Tse2 causes cell death or stasis in recipient cells when delivered by the T6SS. Lacking this information, the physiological role of Tsi2 – the subject of our current study – in the context of an interbacterial interaction was also not known.

**Figure 1:**
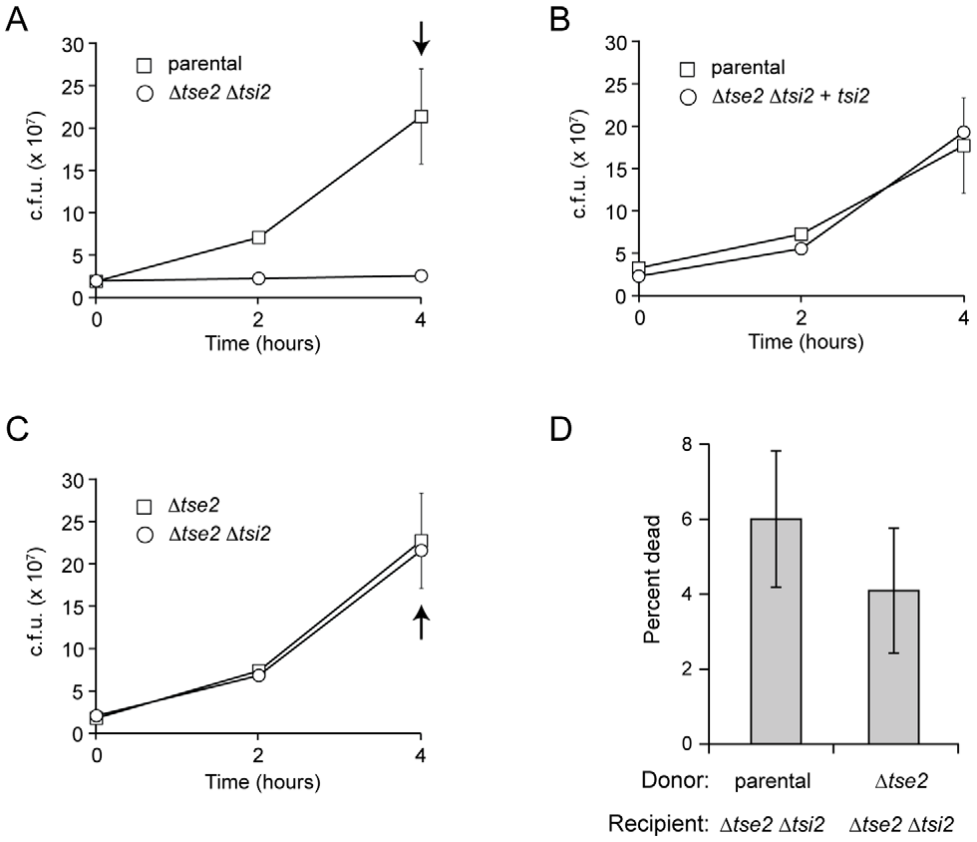
Tsi2 protects against stasis induced by Tse2 delivered via the T6SS. (A–C) Growth competition assays between the indicated donor (squares) and recipient (circles) *P. aeruginosa* strains. For CFU enumeration, strains were distinguished by the presence or absence of LacZ activity. (D) Quantification of dead cells from competition experiments in A and C at the indicated time point (arrow).

To investigate the role of Tsi2 in resisting T6S-dependent Tse2-based intoxication, we monitored changes in donor and recipient CFU during interbacterial competition experiments between *P. aeruginosa* strains. Both donor and recipient strains were generated in the *P. aeruginosa* Δ*retS* background. The deletion of *retS* relieves tight negative posttransciptional regulation of the H1-T6SS and reveals a robust T6S- and Tse2-dependent competitive fitness advantage between strains. Recipient strains bore an additional deletion of the *tse2 tsi2* bicistron, which renders them sensitive to Tse2. Both *tse2* and *tsi2* must be deleted in this strain, as the deletion of *tsi2* alone is lethal in the presence of *tse2*. Donor strains were distinguished from recipients by chromosomal *lacZ* expression from the neutral *attB* site. Interestingly, we found that while total CFU of the donor strain increased exponentially over the course of the competition experiment, CFU of the recipient remained constant (Figure 1A). Consistent with our earlier findings, this inhibition of proliferation required *tse2* in the donor and the absence of *tsi2* in the recipient (Figure 1B & 1C).

We considered three explanations for our finding that the overall population of recipient cells lacking Tse2 immunity did not change during competition experiments against donor cells actively exporting Tse2 by the T6SS: 1) recipient cells are efficiently targeted (approaching 100%) and Tse2 is always bacteriostatic, 2) recipient cells are inefficiently targeted and Tse2 is bactericidal, and 3) recipient cells are efficiently targeted, but differentially affected by Tse2 (unaffected, growth-inhibited, or killed). In the latter two scenarios, the balance between proliferation and death (2), or between proliferation, non-proliferation, and death (3), could produce the stable overall population of recipient bacteria observed. For either of these possibilities, we would expect to observe elevated cell death that is Tse2-dependent in competition experiments between a donor bacterium and a sensitive recipient. However, we found equivalent fractions of dead cells when a sensitive strain was competed against a donor strain capable of delivering Tse2 or one lacking Tse2 (Figure 1D). From these data, we conclude that recipient cells are efficiently targeted by the T6SS, and that the function of Tsi2 is to protects cells from stasis induced by Tse2.

### Tsi2 is stable and directly interacts with Tse2

**Figure 2:**
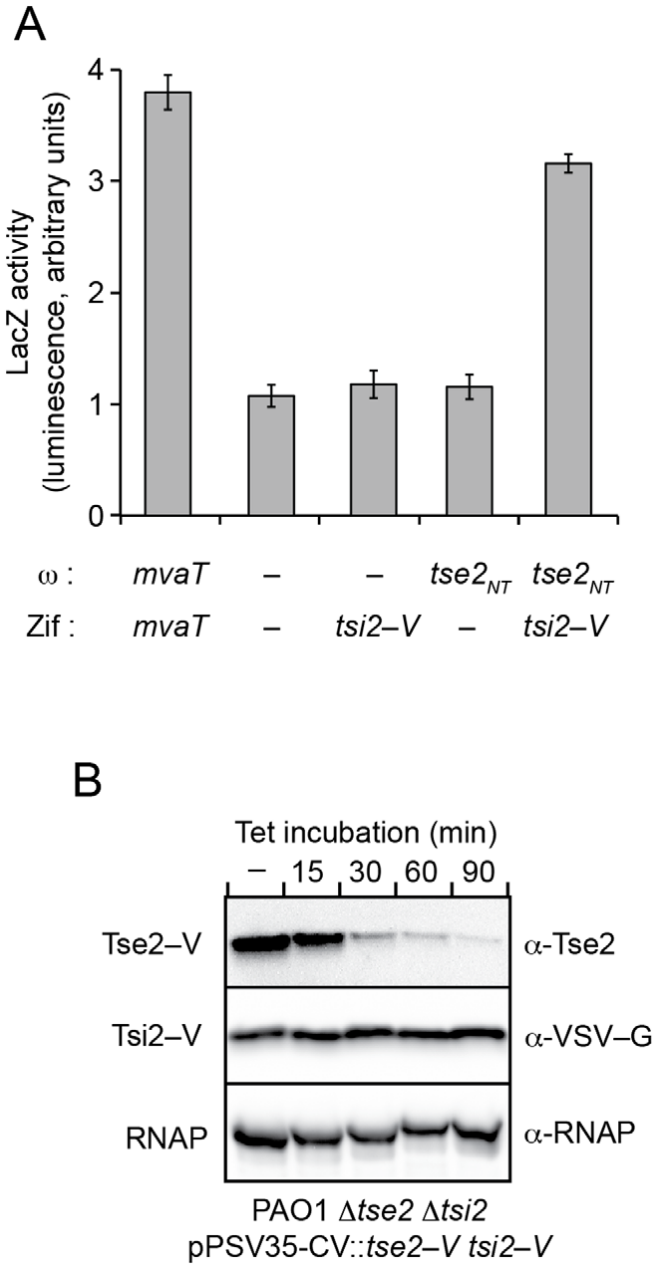
Tsi2 is stable and directly interacts with Tse2. (A) Analysis of Tse2–Tsi2 interaction using the B2H assay. Genes fused in-frame to RNAP–ω and Zif are indicated. The *mvaT* gene codes for a protein previously shown to homo-oligomerize and activate *lacZ* expression in this system. Dashes indicate vectors lacking inserts. A non-toxic allele of tse2 (*tse2* ^T79A/S80A^ (*tse2*_NT_)) was utilized in this and all subsequent B2H assays wherein Tse2 interactions were probed. (B) Western blot analysis of Tse2 and Tsi2 stability in *P. aeruginosa Δtse2 Δtsi2*. The genes were co-expressed in their native bicistronic configuration from the indicated inducible ectopic expression vector. Tetracycline (Tet) was added at time zero to interrupt protein synthesis. Equivalent samples were removed at the indicated times and probed for the presence of each protein. RNA polymerase (RNAP) is included as a loading control.

The substrates of many bacterial secretion pathways require dedicated chaperones for their export. We hypothesized that in addition to its immunity function, Tsi2 might also serve as a dedicated chaperone for Tse2. Although our earlier work has shown that Tse2 and Tsi2 interact in *P. aeruginosa*, whether the proteins bind directly was not determined [13]. As a first step toward investigating the involvement of Tsi2 in Tse2 export, we probed for direct interaction between the proteins using an *E. coli* bacterial two-hybrid (B2H) assay [24]. In the system we employed, fusions of candidate interaction partners are made to a zinc-finger DNA-binding protein, Zif, and the ω subunit of RNA polymerase. Association of the fusion proteins promotes transcription of a *lacZ* reporter gene as described by Dove and colleagues [25]. The broadly toxic nature of Tse2 is a complicating factor for analyzing the protein in heterologous systems such as *E. coli*. Indeed, upon induction of its synthesis from B2H vectors, we found that cellular physiology was rapidly modified, obscuring interpretation of results (data not shown). To facilitate the study of Tse2 in the B2H, we used an allele of the gene encoding a non-toxic Tse2 variant, Tse2^T79A S80A^ (Tse2_NT_). A description of this mutant is provided below. *E. coli* strains expressing Tsi2 C-terminally fused to the vesicular stomatitis virus glycoprotein (VSV-G) epitope followed by the Zif protein (Tsi2–V–Zif) and Tse2_NT_–ω showed significantly enhanced LacZ activity over control strains, indicating that Tsi2 and Tse2 directly interact (Figure 2A).

The interactions between dedicated secretion chaperones and their effector substrates often enhance stability of the effector in the bacterial cytoplasm [26]. However, if Tsi2 behaves analogously to typical antitoxin components of TA modules, even in the absence of stress it would be expected to have a shorter lifetime than the toxin – leaving it unable to act directly in stabilizing Tse2 [27]. Therefore, prior to determining if Tsi2 influences the stability of Tse2, we queried the relative stabilities of the two proteins in *P. aeruginosa*. Western blot analyses of cells following treatment with the protein synthesis inhibitor tetracycline showed an almost complete loss of intact Tse2–V after one hour (Figure 2B). However, no significant decrease in Tsi2–V levels was observed in the cells over the same time period. This was not the result of Tsi2 stabilization by its C-terminal VSV-G fusion, as an N-terminal VSV-G-fused Tsi2 (V–Tsi2) displayed similar stability (Figure S1). The finding that Tsi2 is more stable in cells than its cognate toxin motivated us to further investigate its potential chaperone activity.

### Tsi2 stabilizes Tse2 but is not required for effector secretion

Next we sought to ascertain the influence of Tsi2 on the stability of Tse2 in *P. aeruginosa*. Since Tse2 is toxic in cells lacking Tsi2, this experiment required the use of a non-toxic variant of Tse2. Furthermore, it was necessary that this variant bore only minimal perturbations, such that its stability and overall structure accurately reflected that of the native protein. In light of the additional objective of the study to probe the involvement of Tsi2 in Tse2 secretion, we added the requirement that the non-toxic variant was competent for export through the H1-T6S apparatus.

**Figure 3:**
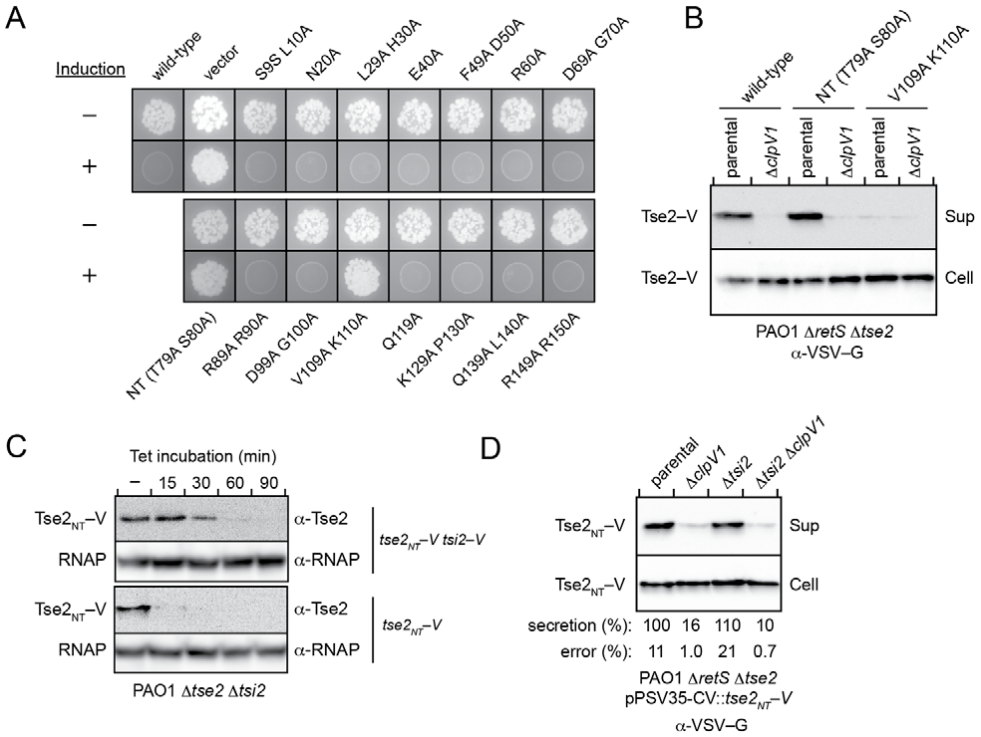
Tsi2 stabilizes Tse2, but is dispensable for Tse2 export by the H1-T6SS. (A) Identification of non-toxic Tse2 double-alanine substitution mutants. Photographs of *P. aeruginosa* Δ*tse2* Δ*tsi2* colonies transformed with an empty vector (pPSV35-CV) or vectors containing the indicated *tse2* alleles grown under inducing (+) or non-inducing (−) conditions. (B) Western blot analysis of wild-type Tse2 and non-toxic Tse2 variants identified in panel A in the cell-associated (Cell) and supernatant (Sup) fractions of the indicated *P. aeruginosa* strains. The *tse2* alleles were ectopically expressed from pPSV35-CV. (C) Analysis of Tse2_NT_ stability in *P. aeruginosa Δtse2 Δtsi2* following the inhibition of protein synthesis by the addition of Tetracycline (Tet) at time zero in the presence (top blots) and absence (bottom blots) of Tsi2. Genes were ectopically expressed from pPSV35-CV. The native *tse2 tsi2* bicistron configuration was utilized for *tse2*_NT_-*tsi2* co-expression. Equivalent samples were removed at the indicated times and probed for the presence of each protein. RNA polymerase (RNAP) is included as a loading control. (D) Western blot analysis of Tse2_NT_ in the cell-associated (Cell) and supernatant (Sup) fractions of the indicated *P. aeruginosa* strains. Values below the blots correspond to average values of secreted Tse2_NT_ based on band densitometry measurements from three independent experiments (% normalized to parental ± standard deviation).

The mechanism of action of Tse2 is not known and our analyses have so far failed to identify sequence motifs that would facilitate the prediction of residues essential for its function. Therefore, we adopted a scanning mutagenesis approach for defining minimal inactivating mutations. Using site-directed mutagenesis, we generated 15 *tse2* alleles encoding adjacent double alanine substitutions at ten amino acid intervals along the length of the protein. In the four cases wherein one of these positions already encoded an alanine, only one substitution was made. Toxicity of the mutant alleles was determined by monitoring the effect of their ectopic expression on the growth of PAO1 Δ*tse2 Δtsi2*. Two alleles, *tse2* ^T79A S80A^ *–V* (*tse2*_NT_ *–V*) and *tse2* ^V109A K110A^ *–V*, displayed a marked decrease in cytotoxicity relative to the wild-type protein (Figure 3A). Western blot analysis showed that the mutant proteins accumulated to levels similar to those of the wild-type protein, suggesting that their lack of toxicity is due to inactivation of the toxin rather than poor expression or decreased stability (Figure S2).

The sequence and structural determinants for effector export by the T6SS remain unresolved; therefore, we proceeded to empirically determine whether the non-toxic Tse2 variants retained H1-T6SS-dependent secretion. Expression plasmids directing the synthesis of Tse2_NT_–V and Tse2^V109A K110A^–V, or Tse2–V were introduced into *P. aeruginosa* Δ*retS* Δ*tse2*. The genes inactivated in this strain lead to constitutive export of effectors by the H1-T6SS (Δ*retS*) and avoid potential competition between native Tse2 and the ectopically-produced protein for the secretory apparatus (Δ*tse2*). As an additional control, we also transformed a plasmid directing the synthesis of Tse2–V into Δ*retS* Δ*tse2 ΔclpV1*. The *clpV1* gene encodes a AAA+ family ATPase that is an important determinant of effector export by the H1-T6SS. Consistent with our earlier finding that Tse2 is a substrate of the H1-T6SS, the wild-type protein was detected in concentrated culture supernatants in a manner dependent on *clpV1* (Figure 3B). The level of secreted Tse2_NT_–V was similar to that of the wild-type protein, whereas secretion of Tse2^V109A K110A^–V was not detected. From these data, we conclude that Tse2_NT_ is a non-toxic substrate of the H1-T6SS.

With a non-toxic and secreted Tse2 mutant in hand, we were able to test the involvement of Tsi2 in Tse2 stability and secretion by the H1-T6SS. The contribution of Tsi2 to Tse2 stability was determined by comparing the lifetime of Tse2_NT_–V when co-expressed in cells with Tsi2–V versus when expressed in cells devoid of Tsi2. For co-expression, Tse2–V and Tsi2–V were produced in their native bicistronic configuration under the control of an inducible promoter. Our data showed that the presence of Tsi2–V significantly extends the lifetime of Tse2_NT_–V in *P. aeruginosa*. In the absence of Tsi2–V, intact Tse2_NT_–V was not detected beyond 15 minutes following the inhibition of protein synthesis; however, it persisted for 60 minutes in the presence of the immunity protein (Figure 3C). The short cellular lifetime of Tse2–V was not due to either its fusion to the VSV-G epitope tag nor to secretion via the H1-T6SS (Figure S1).

To determine the contribution of Tsi2 to Tse2 export by the H1-T6SS, levels of Tse2_NT_–V in culture supernatants from *P. aeruginosa* Δ*retS* strains with or without *tsi2* were compared using quantitative western blotting. This analysis clearly demonstrated that Tsi2 is not required for Tse2 secretion by the H1-T6SS (Figure 3D). Therefore, despite the direct interaction of Tsi2 with Tse2, and the role of this interaction in stabilizing intracellular Tse2, Tsi2 appears to have no role in targeting Tse2 to the secretion apparatus.

### 1.00 Å X-ray crystal structure of a Tsi2 dimer

**Figure 4:**
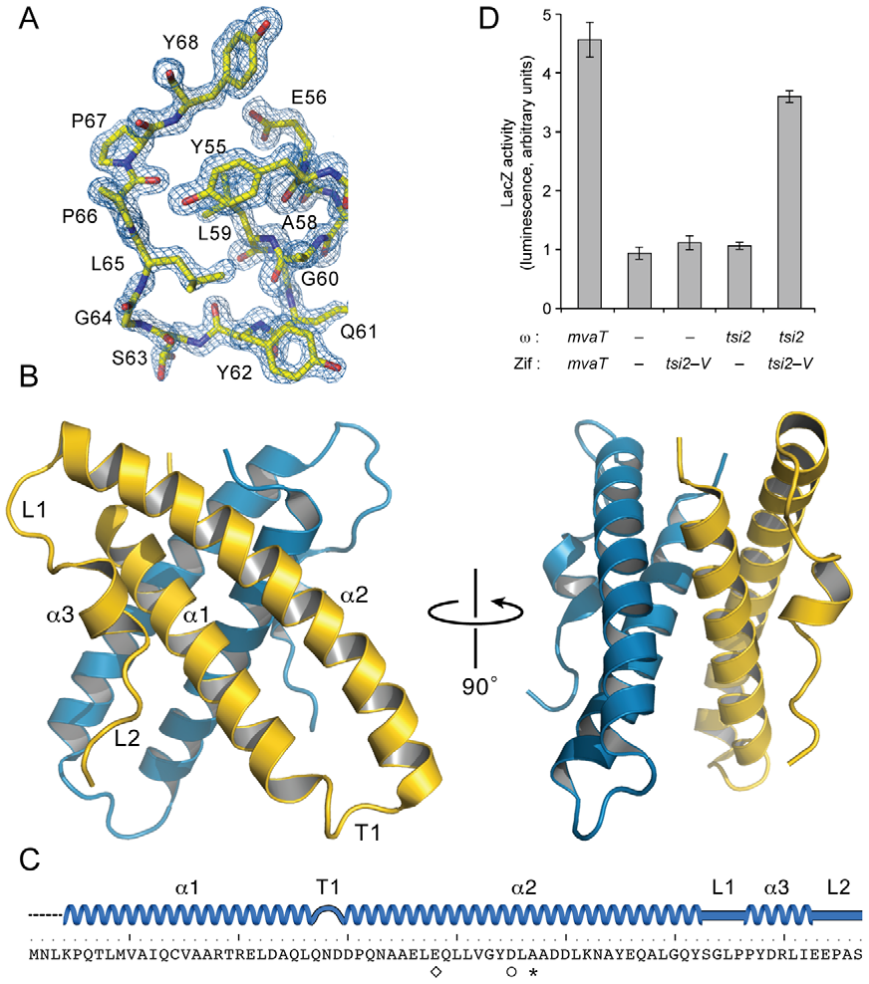
Crystal structure of a Tsi2 dimer. (A) Representative electron density (2*F*_o_−*F*_c_ coefficients) of the Tsi2 structure. Numbers correspond to amino acid positions in the sequence of Tsi2. Residues shown are from Monomer A of the dimer (carbon, yellow; nitrogen, blue; oxygen, red). (B) Ribbon representation of the Tsi2 crystal structure (PDB code: 3RQ9). Protomers of the observed Tsi2 dimer are differentially colored (monomer A, yellow; monomer B, blue) and secondary elements of monomer A are labeled. (C) Schematic depicting the secondary structure of Tsi2. Secondary structure designations defined in the text are provided and key amino acids are highlighted with symbols (diamond, Glu38; circle, Asp45; asterisk, Ala47). (D) B2H analysis of Tsi2 dimerization. Genes fused in-frame to RNAP–ω and Zif are indicated.

To gain additional mechanistic insights into Tsi2 function, we solved its X-ray crystal structure to a resolution of 1.00 Å (Table S1, Figure 4A). Phasing of the structure was obtained using the multiwavelength anomalous diffraction method on a 1.68 Å resolution dataset collected from a crystal containing selenomethionine-substituted, C-terminal hexahistidine-tagged Tsi2 (Tsi2–H_6_) [28]. A molecular model fit to a 1.68 Å resolution electron density map was used to calculate phases for a 1.00 Å data set collected from an isomorphous crystal of native Tsi2–H_6_. Two monomers interacting through extensive contacts were modeled into the crystallographic asymmetric unit (Figure 4B). Each Tsi2 monomer consists of two large α-helices (α1, amino acids 4–26 (based on monomer A); α2, 30–62) arranged as an anti-parallel coiled-coil connected by a short turn (T1, 27–29) (Figure 4C). The remaining C-terminal end of the protein is composed of a short helical segment (α3, 67–72) located between two extended loops (L1 and L2).

In the observed Tsi2 dimer, the long axes of the two monomers are arranged approximately perpendicular to each other and the molecules pack via their coiled-coils. This interaction involves a large (725 Å^2^, 13% total surface area) and predominately hydrophobic (68%) surface area, indicative of a physiologically relevant interface. In agreement with this, the molecular weight of purified Tsi2 measured by gel filtration chromatography was found to closely approximate that of the dimer (calculated, 19.16 kDa; measured, 19.46 kDa), and we observed strong interaction between Tsi2 monomers using the B2H assay (Figure 4D and Figure S3). Superimposition of the Tsi2 monomers showed that overall the two subunits adopt highly similar structures (Cα r.m.s.d, 1.1 Å).

### An acidic surface of Tsi2 mediates interaction with Tse2

As Tse2 has proven recalcitrant to *in vitro* reconstitution, a direct biochemical study of the Tse2–Tsi2 interface has not been feasible. As an alternative strategy, we mutagenized 27 solvent-accessible Tsi2 residues to alanine and probed for effects on toxin immunity as a proxy for functional interaction with Tse2. None of these substitutions, nor a truncation of Tsi2 lacking residues C-terminal of α3, showed a measurable impact on Tse2 interaction (Figure S4). We did not attempt to analyze more extensive truncations of Tsi2, as removing residues from α1 or α2 would likely disrupt its overall fold. From these results we conclude that the interaction of Tse2 with Tsi2 does not require the C-terminal loops and helix of Tsi2, and that interaction is resilient to minor perturbations of the Tsi2 surface.

**Figure 5:**
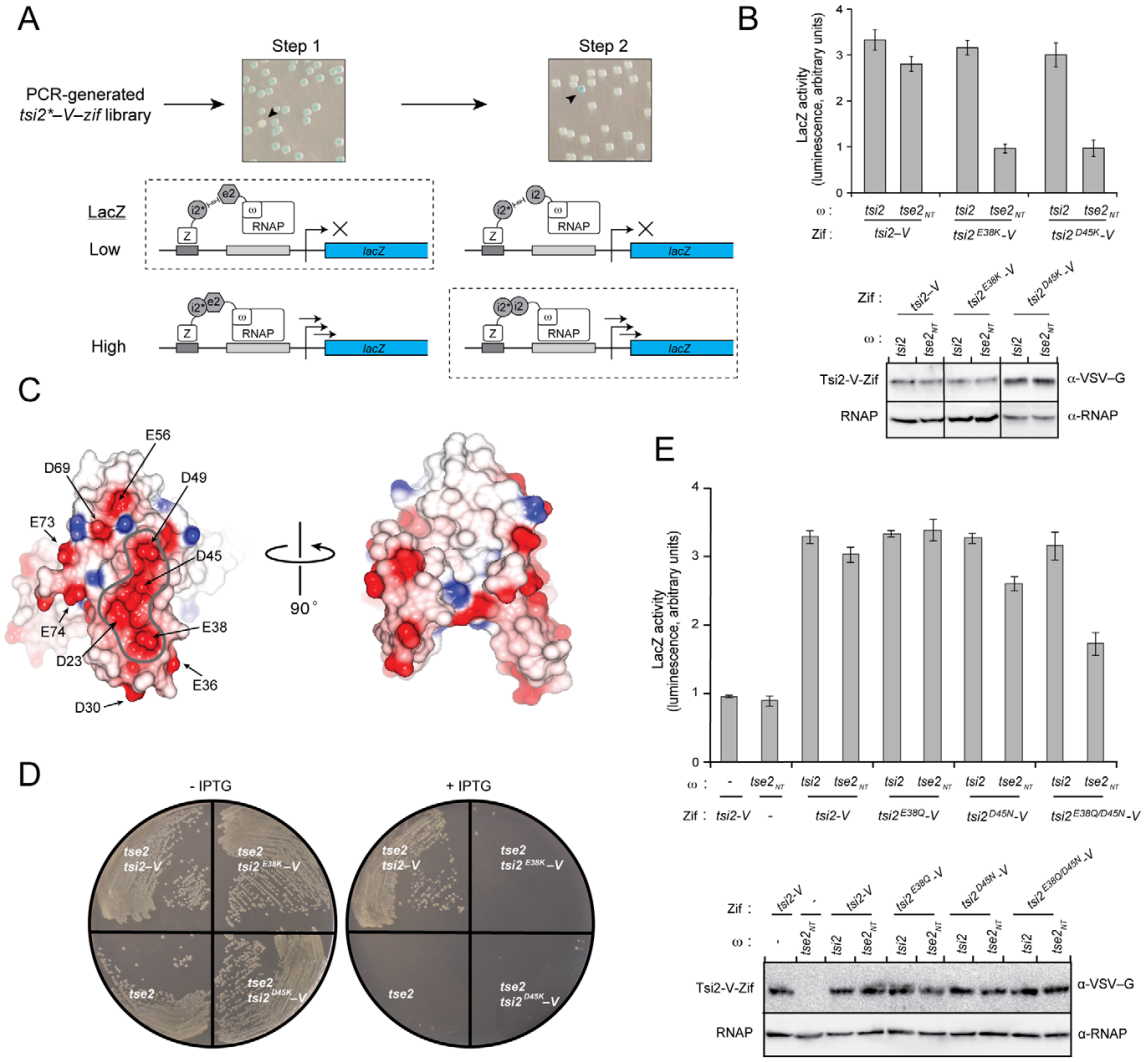
An acidic patch of Tsi2 mediates interaction with Tse2 and is required for toxin immunity. (A) Schematic summarizing the genetic screen conducted to define Tse2 binding determinants of Tsi2. In Step 1, *tsi2* mutants (tsi2*) encoding variants that do not interact with Tse2 are retrieved by selecting colonies of low LacZ activity (arrowhead and dashed box). In Step 2, *tsi2* mutants encoding variants that retain interaction with Tsi2 are retrieved by selecting colonies of containing high LacZ activity (arrowhead and dashed box). (B) Top – B2H analysis of wild-type Tsi2 and Tsi2^E38K^ interaction with Tsi2 and Tse2. Genes fused in-frame to RNAP–ω and Zif are indicated. Bottom – Western blot analysis of the expression of *tsi2–V–zif* alleles within the indicated strains. (C) Molecular surface representation of the Tsi2 dimer colored by electrostatic potential (red, acidic; blue, basic). The border of the acidic patch is outlined for clarity. Acidic surface residues substituted with lysine and tested for Tse2 and Tsi2 binding are indicated. (D) Growth of *E. coli* stains expressing *tse2* alone, or co-expressing *tse2* with the indicated allele of *tsi2–V*, under non-inducing (−IPTG) or inducing (+IPTG) conditions. (E) Top – B2H analysis of conservative Tsi2 acidic patch substitutions on homotypic (Tsi2) and heterotypic (Tse2) interactions. Genes fused in-frame to RNAP–ω and Zif are indicated. Bottom – Western blot analysis of the expression of *tsi2–V–zif* alleles within the indicated strains.

The difficulty we encountered rationally dissecting the Tse2 binding site of Tsi2 led us to pursue a genetic screening strategy. The screen we designed exploited our ability to detect Tsi2 homodimerization and Tse2–Tsi2 association using the B2H (Figure 5A). A random PCR-generated mutant library of *tsi2* was inserted into the pACTR::*V–zif* B2H vector such that clones lacking nonsense mutations would generate N-terminal fusions to V–Zif (Tsi2*–V–Zif). Next, the B2H was used to identify those pACTR::tsi2*–V–zif clones that did not activate *lacZ* expression when co-transformed into cells expressing Tse2_NT_–ω. After cultivation of positive clones, pACTR::tsi2*–V–zif plasmids were isolated, pooled and transformed into cells expressing Tsi2–ω. At this stage, clones of pACTR::tsi2*–V–zif that retained homotypic interaction, and therefore expressed high levels *lacZ* in the presence of Tsi2–ω, were selected for further characterization. While this second stage of our screen was critical for removing major sources of false positives, including *tsi2* nonsense mutations and mutations that abrogated *tsi2* expression, we are also aware of the caveat that it systematically eliminated the potential to recover tsi2* clones that affect both Tse2 and Tsi2 interaction. This issue is addressed below.

Despite screening approximately 20,000 Tsi2* clones, we were able to identify only one single amino acid substitution, Tsi2^E38K^, that specifically abrogated Tse2 interaction when reconstructed and retested in the B2H (Figure 5B). Interestingly, modeling of the electrostatic surface potential of Tsi2 showed that residues in the vicinity of Glu38, including Asp23, Asp45 and Asp49, generate a prominent negatively charged surface feature (Figure 5C). Based on these findings, we hypothesized that this acidic patch on the surface of Tsi2 contributes directly and critically to Tse2 binding. Although our structure of Tsi2 indicates that Glu38 does not mediate intramolecular interactions, we sought to rule-out the possibility that its non-conservative substitution with Lys perturbs native Tsi2 structure and indirectly leads to a loss of Tse2 binding. To this end, we purified Tsi2^E38K^–H_6_ and Tsi2–H_6_ from *E. coli* and compared their secondary structure by circular dichroism spectroscopy (CD). Consistent with our X-ray crystal structure of Tsi2, the CD spectrum of the wild-type protein showed strong helical character (Figure S5). The CD spectrum of Tsi2^E38K^–H_6_ displayed close agreement with the wild-type, suggesting that the Lys substitution does not significantly alter Tsi2 structure (Figure S5).

Tsi2 is a strongly acidic protein (calculated isolectric point, 4.1) with several solvent-exposed negatively charged amino acids located outside of the Glu38-containing acidic surface patch (Figure 5C). To further investigate the specific involvement of this region on Tse2 interaction, we compared the effects of substituting Glu and Asp with Lys within, and outside of, its boundary. In total, we constructed nine additional lysine substitution mutants: three within the acidic patch (D23K, D45K, D49K) and six outside (D30K, E36K, E56K, D69K, E73K, E74K). Using the B2H assay, each Tsi2 substitution mutant was probed for its capacity to both dimerize and associate with Tse2. The nine variants expressed to similar levels as the wild-type protein, and, as expected, none of the substitutions affected Tsi2 dimerization (Figures 5B and S6). Interestingly, while substitutions outside of the acidic patch had no effect on Tse2 interaction, Tsi2 bearing a lysine at position 45 (Tsi2^D45K^), located within the acidic patch, was incapable of binding to Tse2 (Figure 5B). CD spectroscopy confirmed that Tsi2^D45K^–H_6_ retained the secondary structure of the wild-type protein (Figure S5). Surprisingly, no effect on Tse2 binding was caused by the Asp23Lys or Asp49Lys substitutions, suggesting that the residues critical for Tse2 binding within the acidic patch of Tsi2 are Glu38 and Asp45. The observed lack of Tse2 binding by Tsi2^E38K^–V and Tsi2^D45N^–V was also reflected in the immunity properties of the proteins. We observed that both proteins fail to rescue Tse2-based toxicity when expressed in *E. coli* (Figure 5D).

The prominent role of Glu38 and Asp45 in Tse2 binding is further supported by the results of additional mutagenesis studies. Conservative substitutions introduced at these positions displayed a synergistic effect on Tse2 binding. Neither Tsi2 substitutions Glu38Gln nor Asp45Asn alone had a measurable impact on Tse2 interaction, however their combination reduced Tse2 binding by approximately 50%, as determined using the B2H assay (Figure 5E). From these data, we conclude that Glu38 and Asp45 of Tsi2 are major determinants of Tse2 interaction. Furthermore, the substantial loss of Tse2 binding observed upon mutation of these residues to glutamine and asparagine, respectively, suggests that the Tse2–Tsi2 interface is stabilized in part by electrostatic interactions.

### Dimerization of Tsi2 is not required for interaction with Tse2

**Figure 6:**
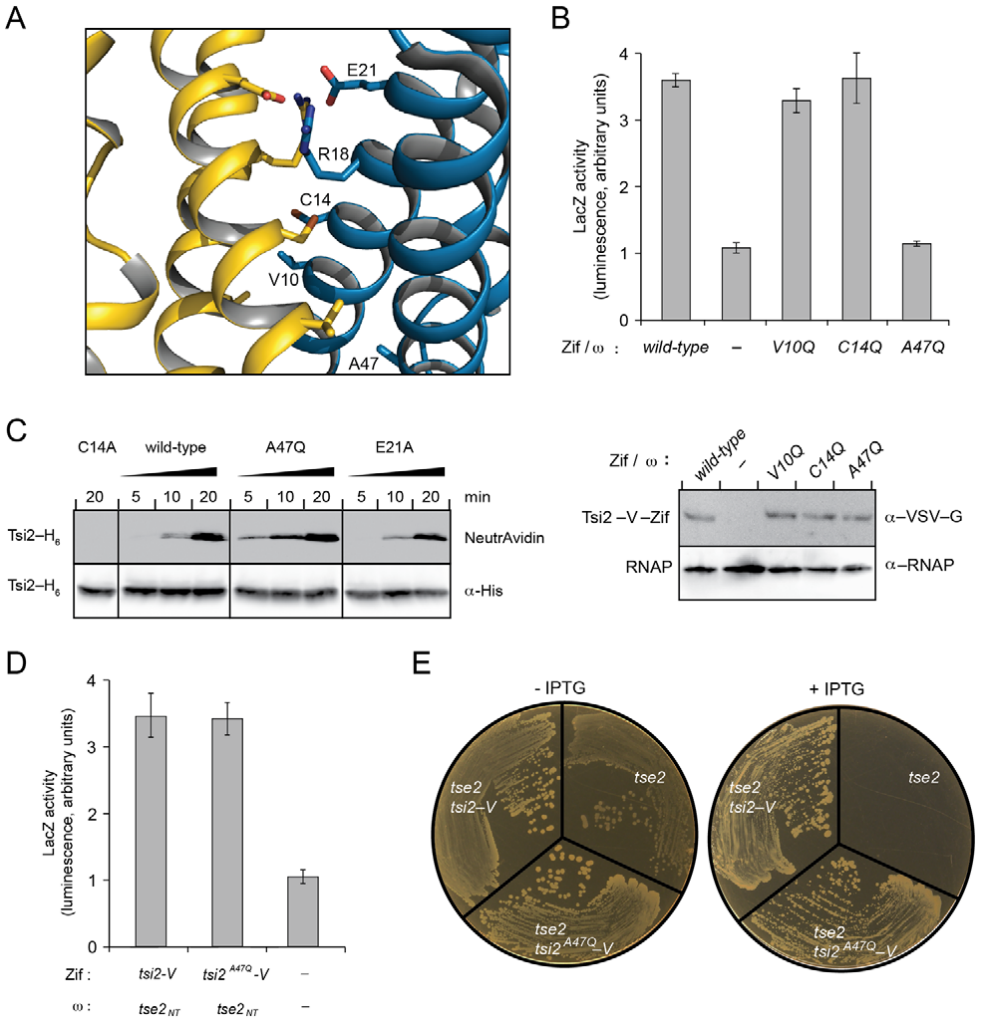
Homodimerization of Tsi2 is not critical for Tse2–Tsi2 interaction. (A) Ribbon representation of the Tsi2 dimer interface. Amino acids positions substituted non-conservatively to disrupt the dimer interface are labeled on monomer A and shown in stick representation. (B) Top – B2H analysis of homodimerization of the indicated Tsi2 dimer interface substitution variants. Genes fused in-frame to RNAP–ω and Zif are indicated. Bottom – Western blot analysis of the expression of *tsi2–V–zif* alleles within the indicated strains. (C) Western blot analysis of Tsi2–biotin–maleimide conjugates. Equal concentration of purified Tsi2 variants (10 µM) were reacted for the indicated time periods with 10 µM of a biotin-maleimide conjugate. (D) B2H analysis of the interaction between wild-type Tsi2 and Tsi2^A47Q^ with Tse2. Genes fused in-frame to RNAP–ω and Zif are indicated. (E) Growth of *E. coli* stains expressing *tse2* alone, or co-expressing *tse2* with the indicated allele of *tsi2*, under non-inducing (−IPTG) or inducing (+IPTG) conditions.

As mentioned above, one caveat of our screen for Tse2-binding determinants of Tsi2 is that it excluded those mutations that also disrupt Tsi2 dimerization. It is conceivable that disruption of the Tsi2 dimer has a generally negative impact on Tse2 binding. Such a scenario could explain the difficulty we encountered in identifying Tsi2 substitutions that lose Tse2, but not Tsi2 interaction. To address this issue, we probed the requirement for Tsi2 dimerization in the interaction of the protein with Tse2. Inspection of the dimer interface permitted the identification of several candidate non-conservative single amino acid substitutions that we predicted could destabilize the Tsi2 dimer. To minimize the probability that our mutations – if successful in disrupting the Tsi2 dimer – would not disrupt Tse2 binding, we limited our mutagenesis to single substitutions. Our initial attempts focused on Arg18 and Glu21, which form a network of polar intersubunit interactions at the origin of the non-crystallographic two-fold rotation axis relating the two subunits in the dimer (Figure 6A). However, B2H analyses of alanine substitution mutants at these positions showed that disruption of this network does not significantly destabilize the overall dimer interface (Figure S7). Together these residues constitute the most significant polar intersubunit contacts, thus we concluded that interfering with hydrophobic interactions would be necessary to disrupt the Tsi2 dimer.

Hydrophobic interactions between Tsi2 monomers are extensive. To increase the likelihood that single amino acid substitutions in non-polar residues at this interface resulted in significant perturbation, we replaced selected residues with the large polar amino acid glutamine. Three spatially distributed small hydrophobic interface residues (all >90% solvent inaccessible), Val10, Cys14, and Ala47, were selected, substituted with glutamine, and tested for homodimerization (Figure 6A). Of the three mutant proteins, only Tsi2^A47Q^ displayed decreased activity in the B2H (Figure 6B). Levels of this protein were similar to the other mutant proteins and the wild-type, consistent with the diminished activity observed specifically resulting from a failure to efficiently homodimerize. As an independent measure of dimer formation by Tsi2^A47Q^, we employed an *in vitro* cysteine accessibility assay. This assay is based on our observations that Tsi2 possesses only one cysteine residue, and that this amino acid is solvent inaccessible at the dimer interface (Figure 6A). Destabilization of the interface is expected to increase the reactivity of the Cys14 sulfhydryl side chain to small soluble maleimide-containing probes. To test for differential reactivity at this site, we purified Tsi2–H_6_, Tsi2^A47Q^–H_6_, and Tsi2^C14A^–H_6_ and Tsi2^E21A^–H_6_ as controls, and incubated the proteins with biotin-maleimide. Reactions were separated by SDS-PAGE and protein-biotin-maleimide conjugates were visualized by probing with Neutravidin-HRP. Consistent with our B2H data, Tsi2^A47Q^–H_6_ reacted more rapidly than the wild-type protein or Tsi2^E21A^–H_6_ (Figure 6C). Tsi2^C14A^–H_6_ displayed no observable reactivity under these conditions, indicating that the products observed were specifically due to the reactivity at this site. Taken together with our B2H data, these data strongly suggest that the native Tsi2 dimer interface is significantly disrupted in Tsi2^A47Q^.

With a Tsi2 dimer-defective variant in hand, we next sought to measure the impact of dimer disruption on heterotypic interactions of Tsi2 with Tse2. First, using the B2H assay, we observed no significant difference between Tsi2–V and Tsi2^A47Q^–V binding with Tse2 (Figure 6D). As a second, functional measure of Tse2–Tsi2 interaction, we also tested the capacity of Tsi2^A47Q^ to provide immunity to Tse2. Our data showed that Tsi2^A47Q^–V, like Tsi2–V, provides full protection against Tse2-based toxicity in *E. coli* (Figure 6E). In total, these data show that the Tse2–Tsi2 interaction is insensitive to the oligomeric state of Tsi2. Taken together with our screening data, we conclude that the regions of Tsi2 important for Tsi2 homotypic versus Tse2 heterotypic interactions are topologically distinct; Tse2 binding occurs at the face of Tsi2 opposite the site of homodimerization.

## Discussion

This study has shown that the Tse2–Tsi2 system has a unique set of properties that do not neatly conform to existing paradigms for toxin-antitoxin and effector-chaperone systems. For example, unlike canonical antitoxins, Tsi2 is more stable than its cognate toxin. This may reflect different physiological functions of the two systems. While the roles of TA systems are variable, and in certain instances remain a matter of debate, it is well established that they serve important functions in gene maintenance and response to stress [20]. For both of these functions, the activity of the TA system involved is mediated by modulation of toxin activity through antitoxin degradation. Our finding that Tsi2 stability greatly exceeds that of Tse2 suggests that this system has not evolved to conditionally release the toxin. Therefore, assuming an adequate expression level, *P. aeruginosa* strains endowed with *tsi2* are likely to possess stable, non-dynamic immunity to growth inhibition by Tse2. In this way, Tsi2 is more akin to certain colicin immunity proteins, which bind their cognate toxin with extraordinary affinity and provide complete protection against both endogenous and xenogenous cognate toxin [17], [29].

Despite the functional disparities between the Tse2–Tsi2 and TA systems, they do possess notable parallels. One common property of TA systems is that the components have strongly opposing electrostatic character [19]. In the majority of instances, the antitoxin is more acidic than its cognate toxin. This is also the case for the Tse2–Tsi2 system, wherein Tsi2 is highly acidic and the difference in the calculated isoelectric points of the two proteins is 2.6. This analogy holds, and is even more striking, when one considers the other Tse-Tsi pairs. The differences in toxin and immunity isoelectric points are greater in magnitude for these proteins (Tse/i1, 4.1; Tse/i3, 3.6), and in both cases the immunity proteins are acidic. A second shared physical attribute of TA and the Tse2–Tsi2 systems is that their antitoxin and immunity components, respectively, display modularity in their homotypic and heterotypic interactions. Although type II antitoxins are highly diverse at the sequence level, recent biochemical and structural analyses of these proteins indicate that they often exist as dimers and, despite their small size, homomeric contacts occur at a site physically removed from the site of cognate toxin interaction [30]. Our discovery that amino acids positions of Tsi2 critical for Tse2-binding reside on the face of the protein opposite from those involved in its homodimerization, taken together with our ability to readily generate specific loss-of-function mutations at either of these sites, strongly suggest an analogous configuration of the Tse2–Tsi2 complex.

We found that co-expression of *tsi2* with *tse2* leads to a significant increase in the stability of the toxin, suggesting that the two proteins closely interact. Despite this, cells lacking Tsi2 have no detectable defect in Tse2 secretion, indicating that Tsi2 does not–in addition to its immunity properties–play a role in targeting Tse2 to the secretion apparatus. The specialization of Tsi2 as an immunity protein is in line with our current understanding of the function of other T6SS effector immunity proteins, Tsi1 and Tsi3. These proteins reside in the periplasm and therefore are unavailable to assist in ushering their cognate toxins to the H1-T6SS [14]. This leaves open the question of how T6S effectors are recognized by the apparatus. One possibility is that effectors are bound by yet unidentified specialized secretion chaperone(s). In this case, such a protein might remove Tse2 from the Tse2–Tsi2 complex prior to export. Since we observed no impact of Tsi2 on Tse2 secretion, we would expect that such a protein would either bind Tse2 with higher affinity than Tsi2, or that it would bind a region of Tse2 not involved in Tsi2 binding and target the protein to the secretion machine, where Tsi2 would be readily removed.

An alternative explanation for our finding that Tsi2 has no impact on Tse2 export is that the Tse proteins are exported co-translationally. In this model, Tsi2, like Tsi1 and Tsi3, might be present largely to protect against cognate Tse proteins arriving *in trans* via the T6SSs of adjacent bacteria. Co-translational export of the Tse proteins could also help reconcile how periplasmic effectors, in particular Tse1, which possess numerous cysteine residues, avoid misfolding in the reduced cytoplasmic environment. Based on this model, one would predict that the H1-T6SS and its effectors would be tightly co-regulated. In *P. aeruginosa*, expression of *tse* and HSI-I genes (encode the H1-T6S apparatus) are coordinately co-regulated by the Gac/Rsm pathway [7], [31]. Interestingly, this pathway stringently controls expression at the posttransciptional level, which would appear logical if a build-up of cytoplasmic effector was undesirable.

**Figure 7:**
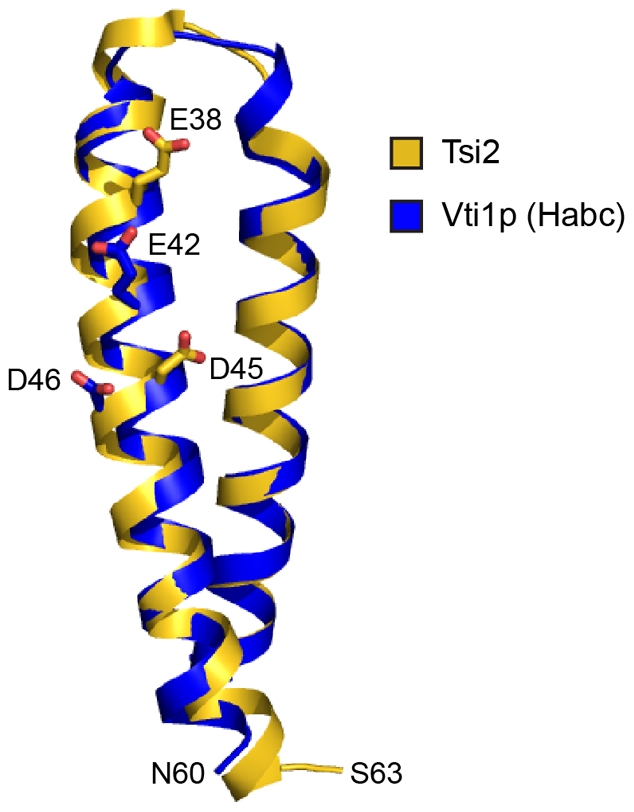
Tsi2 shares structural similarity with the N-terminal Habc domain of the Vti1p SNARE protein. Superimposition of the N-terminal Habc domain (blue; residues 3–60, helix c not shown for clarity) with the coiled coil region of Tsi2 (yellow; residues 3–63). Acidic amino acids implicated in heterotypic protein interactions are depicted using stick representation.

Teng and colleagues recently reported the crystal structure of the N-terminal three-helix bundle (Habc) domain of the yeast SNARE (soluble N-ethylmaleimide-sensitive factor activating protein receptor) Vti1p bound to its epsin-like adaptor protein Ent3p [32]. According to analyses using DALI, the Vti1p Habc domain is the most similar structure to Tsi2 in the current protein databank (Z score, 7.8; Cα r.m.s.d, 1.2 Å). Specifically, a close match of the length and curvature of the two large helices of Tsi2 to helices A and B of the Habc domain leaves these regions of the two structures nearly indistinguishable (Figure 7). Not only are these proteins structurally related, they also appear to interact with binding partners in a spatially and chemically similar fashion. Two acidic residues located on helix B of the Habc domain, Glu42 and Asp46, were identified by Teng and colleagues as critical determinants of Ent3p binding. Substitution of either residue with arginine severely disrupted the interaction of Vti1p with Ent1p and led to mislocalization of Vti1p in yeast [32]. Interestingly, in an overlay of the two structures, these residues are found in close proximity to the acidic residues of Tsi2 discovered in our study to mediate Tse2 interaction (Figure 7). Although the simple structure of Tsi2 necessarily reduces confidence in interpreting the significance of structural similarity to the protein, we find the extent of structural and functional similarity between Tsi2 and the Habc domain striking. Our overall limited understanding of T6S makes it difficult to reconcile the relatedness of Tsi2 to the Habc domain, however it is worth noting that Tsi2 is now the second T6S protein shown to possess structural homology to an N-terminal regulatory domain of a SNARE protein. We recently reported that TagF, a negative posttranslational regulator of the T6SS, displays significant similarity to the N-terminal longin domain of Sec22b [33]. This domain has no structural homology to the Habc domain, however both can function analogously in directing subcellular localization by mediating interactions with adaptor proteins [34]. It will be of interest to determine the evolutionary mechanisms underlying the relationship between Tsi2 and TagF and proteins involved in vesicle trafficking.

The structure of Tsi2 marks an important first step toward a complete molecular characterization of the Tse2–Tsi2 T6S toxin-immunity pair. However, many key outstanding questions remain. Foremost among these remains the mechanism of action of Tse2. Our analysis of the effects of Tse2 on *P. aeruginosa* cells during intraspecies competition suggests that the protein acts efficiently and specifically to cease growth, and avoid killing targeted cells. Such effects have been observed for TA system toxins that act by cleaving mRNA, such as RelE [35]. Strong evidence that Tse2 also functions as a ribonuclease is lacking, however there are noteworthy indications. For example, the Phyre (Protein Homology/AnalogY Recognition Engine) structure prediction algorithm reports similarity between Tse2 and enzymes that bind and hydrolyze nucleic acids [36]. We found that an acidic patch of amino acids located on the surface of Tsi2 mediates interaction with, and immunity against Tse2. If like most antitoxins, Tsi2 inhibits its cognate toxin by active site occlusion, it is conceivable that the negatively charged character of Tsi2 could engage basic residues of Tse2 that would otherwise participate in nucleic acid binding [37].

## Materials and Methods

### Plasmid and strain construction

*Pseudomonas aeruginosa* strains used in this study were derived from the sequenced strain PAO1 [38]. *P. aeruginosa* strains were grown on Luria-Bertani (LB) media at 37°C supplemented, when appropriate, with 30 µg/ml gentamycin, 25 µg/ml irgasan, 40 µg/ml X-gal, and stated concentrations of Isopropyl β-D-1-thiogalactopyranoside (IPTG). *Escherichia coli* strains used in this study included DH5α for plasmid maintenance, SM10 for conjugal transfer of plasmids into *P. aeruginosa*, and BL21 pLysS for expression of Tse2 and Tsi2. The *tse2*, *tsi2* genes and *tse2 tsi2* and *tse2–vsv-g tsi2* bicistronic sequences were PCR-amplified from *P. aeruginosa* genomic DNA and cloned into pPSV35CV [13], pET29b+ and pET21a+ vectors (Novagen). Site-directed mutants of *tsi2* and *tse2* were generated using either QuickChange (Stratagene) or Kunkel mutagenesis procedures [39]. Chromosomal fusions and in-frame gene deletions were generated as described previously and were verified by DNA sequencing [7], [40]. The ΔHSI-I strain was constructed such that all sequence between nucleotide 2015 of PA0074 (*ppkA*) and nucleotide 754 of PA0091 (*vgrG1*) were deleted.

### Growth competition assays

*P. aeruginosa* cultures were grown overnight at 37°C in LB broth containing 0.01% L-arabinose. In each experiment, the donor strain contained *lacZ* inserted at the neutral phage attachment site [41]. LacZ-labeled donor and non-labeled recipient strains were mixed at a ratio of 1∶1 and spotted onto a 0.2 µm nitrocellulose membrane (Whatman) on a 3% LB agar plate containing 0.2% L-arabinose. Competitions were incubated at 37°C and harvested at the indicated time points by resuspending bacterial cells in LB and plating onto LB plates containing 40 µg/ml X-gal for CFU enumeration.

### Live/dead staining of bacterial cells

Growth competition assays of *P. aeruginosa* Δ*retS* and Δ*retS* Δ*tse2* against *P. aeruginosa* Δ*retS* Δ*tse2* Δ*tsi2* were performed on filters as described above. At 4 hrs after initiating the experiment, the filters were removed from agar plates and resuspended in 3 ml LB broth. The cells were collected by centrifugation, washed once with 1× phosphate buffered saline (PBS) and resuspended in 100 µl PBS. The bacterial suspension was stained with the LIVE/DEAD BacLight Bacterial Viability Kit (Molecular Probes) according to the protocol of the manufacturer. Viability was measured using a fluorescence microscope equipped with FITC and mCherry filters. The ratio of live/dead cells was determined by calculating the green/red fluorescent cells for 12 random fields per competition. Three independent experiments were performed.

### Protein expression and purification

C-terminal hexahistidine-tagged Tsi2 (Tsi2–H_6_) proteins were overexpressed in *E. coli* BL21 pLysS. Overnight cultured cells were back-diluted 1∶1000 into fresh 2× Yeast Tryptone (YT) media or defined SelenoMet medium base and SelenoMet nutrient mix medium (Athena Enzyme Systems). Expression was induced at an OD_600_ of 0.5 with 0.1 mM IPTG for 16 hrs. at 20°C. Cells were harvested by centrifugation (8000× g; 20 min, 4°C) and resuspended in lysis buffer [50 mM Tris-HCl, pH 7.5, 0.5 M NaCl, 1% (v/v) Triton X-100, 5% (v/v) glycerol, 1 mM DTT, and protease inhibitor cocktail (Roche Diagnostics)]. Tsi2–H_6_ was purified by affinity chromatography using a HisTrap FF column (GE Healthcare) followed by size-exclusion chromatography on a HiPrep 16/60 Sephacryl S-200 high-resolution column (GE Healthcare) using the AKTA Explorer FPLC System. Purified proteins were stored in a buffer containing 50 mM Tris-HCl pH 7.5, 500 mM NaCl, 1 mM DTT, and 5% (w/v) glycerol and dialyzed into a buffer containing 5 mM Tris-HCl pH 7.5, 5 mM NaCl, and 1 mM DTT for crystallization.

### Crystallization, structure solution, and structure analysis

Crystals of Tsi2-H_6_ were grown at 25°C by hanging drop vapor diffusion. An equal volume of 10 mg/ml protein sample was mixed with the crystallization solution (0.1 M sodium acetate, pH 5.0 and 8% polyethylene glycol (PEG) 4000). Crystals were cryo-protected in reservoir solution containing 25% PEG 4000 and flash frozen in liquid nitrogen. Diffraction data were collected at the Lawrence Berkeley National Laboratory Advanced Light Source (ALS) Beamline 8.2.1 (University of California, Berkeley). Data were reduced using HKL2000 [42]. Phases were obtained experimentally with data obtained from selenomethionine-substituted Tsi2-H_6_ for structure solution by multi-wavelength anomalous dispersion (MAD) using the SOLVE program [43]. The final model was built by iterative model building and maximum likelihood refinement with Refmac-5 [44]. Finally, 123 well-defined water molecules were added, and refinement was continued until the R-value converged to 0.144 (R_free_ = 0.176) for all reflections to 1.00 Å resolution. The CCP4 [45] suite and XtalView [46] were used for crystallographic calculations. Molecular figures were generated with PyMOL [47] and CCP4 Molecular Graphics [48]. The model was validated using MolProbity [49]. All residues in the final model lie within allowed regions of a Ramachandran plot and 99.4% lie within the Ramachandran favored region. The crystal structure and structure factors have been deposited in the Protein Data Bank (PDB entry 3RQ9) [50].

### Bacterial two-hybrid analysis

Tse2 and Tsi2 derivatives were cloned into pBRGP–ω and pACTR–*V–zif* plasmids [25], [51]. The pBRGP::*tsi2*–ω and pBRGP::*tse2_NT_*–ω derivatives direct the synthesis of Tsi2 or Tse2 wild-type and mutant alleles as N-terminal fusions to the ω subunit of *E coli* RNAP. Plasmid pACTR::*tsi2– V–zif* directs the synthesis of the Tsi2-VSV-G fusion to the N-terminus of the zinc finger DNA-binding domain of murine Zif268 (Zif). The *tsi2* gene was mutagenized randomly by PCR with Taq DNA polymerase. A pool of plasmids encoding the resulting *tsi2* mutants were ligated into the pACTR– *V–zif* plasmid and transformed into DH5α-F′IQ cells. All resulting transformants were pooled for plasmid isolation. Pooled plasmids were co-transformed with pBRGP::*tse2_NT_*–ω into KDZif1ΔZ competent cells and plated onto LB plates containing 12.5 µg/ml tetracycline, 150 µg/ml carbenicillin, 50 µg/ml kanamycin, 40 µg/ml X-gal, and 500 µM Phenylethyl-β-D-galactosidase (tPEG). LacZ negative (white) colonies were selected for inoculation into 96 well plates, subcultured three times to cure plasmid pBRGP::*tse2_NT_*–ω and pooled for plasmid isolation yielding pACTR::tsi2*–V–zif. Purified plasmids were digested by ScaI and T7 exonuclease for removal of pBRGP::*tse2*_NT_–ω. After purification, the mutated pACT::tsi2*–V–zif plasmids were co-transformed with pBRGP::*tsi2*–ω into KDZif1ΔZ competent cells and transformants were plated onto LB plates containing 12.5 µg/ml tetracycline, 150 µg/ml carbenicillin, 50 µg/ml kanamycin, 40 µg/ml X-gal and 500 µM tPEG. LacZ positive (blue) colonies were subcultured and subjected to plasmid isolation and subsequent sequencing analysis.

### β-Galactosidase assays

*E. coli* KDZif1ΔZ cells were grown to an OD_600_ of 1.0, permeabilized with 10% CHCl_3_, and β-galactosidase activity was quantitatively assayed using a Galacto-Light Plus kit as previously described [52]. Assays were performed with at least two individual experiments in triplicate. Representative data sets are shown and values consist of averages based on three independent measurements from one experiment.

### Preparation of proteins and western blotting

Cell associated and supernatant protein samples were prepared as previously described [7]. Western blotting was performed as described previously using α-VSV-G, α-Tse2 and α-RNA-polymerase, with the modification that α-VSV-G antibody probing was performed in 5% BSA in Tris-buffered saline containing 0.05% v/v Tween 20 [14]. HisProbe-HRP Kit was used for direct detection of recombinant His-tagged proteins according to the manufacturer's instructions (Thermo Scientific).

### Bacterial growth analyses

For growth curves, *E. coli* BL21 pLysS cells harboring pET29b+ expressing Tse2 and Tsi2 derivatives were grown overnight in liquid LB broth at 37°C and back-diluted into LB broth (1∶1000) supplemented with 50 µg/ml kanamycin and 12.5 µg/ml chloramphenicol. Cultures were grown to an OD_600_ of 0.1–0.2 and induced with 0.2 mM IPTG. OD_600_ measurements were determined for *E. coli* strains in LB broth using an automated BioScreen C Microbiology plate reader with agitation at 37°C. Three independent measurements were performed in triplicate for each strain. A *VSV-G* epitope sequence was fused to *tsi2* to allow for analyzing Tsi2 expression. For growth on solid medium, *E. coli* BL21 pLysS cells expressing Tse2 and Tsi2 derivatives were grown on LB agar plates with or without IPTG induction.

### Circular dichroism spectroscopy

CD spectra were recorded on a Jasco J810 Circular Dichroism Spectrometer using a 1 mm path-length quartz cuvette (Starna). Tsi2 proteins were measured in triplicate at 195–260 nm in 1× PBS buffer, pH 7.5 at 25°C. A total of three scans were recorded and averaged for each spectrum.

### In vitro labeling with biotin-maleimide

Purified proteins were exchanged into a 1× PBS buffer containing 50 mM NaCl, pH 7.5. Biotin-maleimide was solubilized in Dimethyl sulfoxide (DMSO) and added to the protein samples at a final concentration of 10 µM. Protein samples (10 µM) were incubated with biotin-maleimide at room temperature and reactions were quenched at indicated time points by the addition of a final concentration of 0.1 mM Tris, pH 8.0. Western blots were used to detect biotin-maleimide labeled Tsi2 with NeutrAvidin and to detect His-tagged Tsi2 with HisProbe-HRP Kit.

### Analytical gel filtration

Purified Tsi2 was loaded onto a Superdex-200 10/300GL HR10/30 column (GE Healthcare) equilibrated with a buffer containing 50 mM Tris pH 7, 500 mM NaCl, and 5% glycerol. Protein standards included ribonuclease A (13700 Da), carbonic anhydrase (29000 Da), ovalbumin (43000 Da), conalbumin (75000 Da), and aldolase (158000 Da).

### Protein half-life determination

*P. aeruginosa* Δ*tse2*Δ*tsi2* strains harboring pPSV35::*tse2_NT_–V*, pPSV35::*tse2–V tsi2*–*V* or pPSV35::*tse2_NT_–V tsi2*–*V* were grown at 37°C with aeration in LB broth containing 30 µg/ml gentamycin. Overnight cultures were back-diluted 1∶500 into LB containing 30 µg/ml gentamycin and 0.5 mM IPTG. After *P. aeruginosa* cells were grown at 37°C for 5 hrs., protein synthesis was inhibited with the addition of 250 µg/ml tetracycline. Samples were taken at indicated time points and analyzed by Western blot.

## Supporting Information

Figure S1 **Epitope tags and export via the H1-T6SS do not account for the Tsi2 and Tse2 stability observed in** **Figures 2B** **and** **3C** **, respectively.** Analysis of Tse2_NT_ and V–Tsi2 stability in *P. aeruginosa Δ*HSI-I *Δtse2 Δtsi2* following the inhibition of protein synthesis by the addition of tetracycline (Tet). Top blot – the stability of untagged Tse2_NT_ is similar to that observed for the C-terminally VSV–G fused protein (Fig. 3C). Bottom blot – N-terminally VSV–G fused Tsi2 (V–Tsi2) displays similar stability to the C-terminally-tagged protein (Fig. 2B). These experiments were conducted in an HSI-I deletion strain in order to rule out Tse2 secretion as a factor contributing to its depletion from cells.
(TIF)

Figure S2 **Non-toxic Tse2 alleles express to similar levels as the wild-type.** Western blot analysis of the indicated *tse2–V* alleles expressed in *P. aeruginosa Δtse2Δtsi2* grown on LB agar plates containing 30 µg/µl gentamycin and 0.5 mg/ml IPTG. RNA polymerase (RNAP) was included as a loading control.
(TIF)

Figure S3 **Analytical gel filtration elution profile of the Tsi2 dimer.** Purified Tsi2–H_6_ was loaded onto a Superdex-200 10/300GL HR10/30 column (GE Health Care). Standard curves were calculated using linear regression analysis.
(TIF)

Figure S4 **Tsi2 surface amino acid alanine substitutions do not affect its function.** Growth of *E. coli* BL21 pLysS cells harboring pET29b+ co-expressing *tse2* with the indicated *tsi2–V* alleles. Growth was measured in LB broth containing kanamycin using the automated BioScreen C Microbiology plate reader with agitation at 37°C. Bars represent growth normalized to *tsi2* wild-type at six hours post-inoculation. Error bars represent standard deviation of three independent measurements.
(TIF)

Figure S5 **Substitution of Tsi2 acidic patch residues with lysine does not significantly alter structure.** Far-UV CD spectrum of Tsi2–H_6_, Tsi2^E38K^–H_6_ and Tsi2^D45K^–H_6_ at 20°C.
(TIF)

Figure S6 **Tsi2 surface acidic residue lysine substitution mutants that do not impact homodimerization or interaction with Tse2.** B2H analysis of Tsi2 acidic residue substitutions on homotypic (Tsi2) and heterotypic (Tse2) interactions. Genes fused in-frame to RNAP–ω and Zif are indicated.
(TIF)

Figure S7 **Nonconservative substitutions of Cys14 and polar dimer interface residues do not impact Tsi2 dimer formation.** B2H analysis of Tsi2 dimer interface amino acid substitutions on Tsi2 dimerization. Genes fused in-frame to RNAP–ω and Zif are indicated.
(TIF)

Table S1 **Tsi2 data collection, phasing and refinement statistics.**
(PDF)

## References

[1] Cascales E (2008). The type VI secretion toolkit.. EMBO Rep.

[2] Schwarz S, Hood RD, Mougous JD (2010). What is type VI secretion doing in all those bugs?. Trends Microbiol.

[3] Alvarez-Martinez CE, Christie PJ (2009). Biological diversity of prokaryotic type IV secretion systems.. Microbiol Mol Biol Rev.

[4] Cornelis GR (2006). The type III secretion injectisome.. Nat Rev Microbiol.

[5] Boyer F, Fichant G, Berthod J, Vandenbrouck Y, Attree I (2009). Dissecting the bacterial type VI secretion system by a genome wide in silico analysis: what can be learned from available microbial genomic resources?. BMC Genomics.

[6] Leiman PG, Basler M, Ramagopal UA, Bonanno JB, Sauder JM (2009). Type VI secretion apparatus and phage tail-associated protein complexes share a common evolutionary origin.. Proc Natl Acad Sci U S A.

[7] Mougous JD, Cuff ME, Raunser S, Shen A, Zhou M (2006). A virulence locus of Pseudomonas aeruginosa encodes a protein secretion apparatus.. Science.

[8] Veesler D, Cambillau C (2011). A common evolutionary origin for tailed-bacteriophage functional modules and bacterial machineries.. Microbiol Mol Biol Rev.

[9] Kanamaru S (2009). Structural similarity of tailed phages and pathogenic bacterial secretion systems.. Proc Natl Acad Sci U S A.

[10] Bingle LE, Bailey CM, Pallen MJ (2008). Type VI secretion: a beginner's guide.. Curr Opin Microbiol.

[11] Aschtgen MS, Bernard CS, De Bentzmann S, Lloubes R, Cascales E (2008). SciN is an outer membrane lipoprotein required for Type VI secretion in enteroaggregative Escherichia coli.. J Bacteriol.

[12] Weber B, Hasic M, Chen C, Wai SN, Milton DL (2009). Type VI secretion modulates quorum sensing and stress response in Vibrio anguillarum.. Environ Microbiol.

[13] Hood RD, Singh P, Hsu F, Guvener T, Carl MA (2010). A type VI secretion system of Pseudomonas aeruginosa targets a toxin to bacteria.. Cell Host Microbe.

[14] Russell AB, Hood RD, Bui NK, LeRoux M, Vollmer W (2011). Type VI secretion delivers bacteriolytic effectors to target cells.. Nature.

[15] Stebbins CE, Galan JE (2003). Priming virulence factors for delivery into the host.. Nat Rev Mol Cell Biol.

[16] Aoki SK, Pamma R, Hernday AD, Bickham JE, Braaten BA (2005). Contact-dependent inhibition of growth in Escherichia coli.. Science.

[17] Cascales E, Buchanan SK, Duche D, Kleanthous C, Lloubes R (2007). Colicin biology.. Microbiol Mol Biol Rev.

[18] Hayes CS, Aoki SK, Low DA (2010). Bacterial contact-dependent delivery systems.. Annu Rev Genet.

[19] Yamaguchi Y, Inouye M (2011). Regulation of growth and death in Escherichia coli by toxin-antitoxin systems.. Nat Rev Microbiol.

[20] Gerdes K, Christensen SK, Lobner-Olesen A (2005). Prokaryotic toxin-antitoxin stress response loci.. Nat Rev Microbiol.

[21] Maisonneuve E, Shakespeare LJ, Jorgensen MG, Gerdes K (2011). Bacterial persistence by RNA endonucleases.. Proc Natl Acad Sci U S A.

[22] Yamaguchi Y, Inouye M (2009). mRNA interferases, sequence-specific endoribonucleases from the toxin-antitoxin systems.. Prog Mol Biol Transl Sci.

[23] Ramage HR, Connolly LE, Cox JS (2009). Comprehensive functional analysis of Mycobacterium tuberculosis toxin-antitoxin systems: implications for pathogenesis, stress responses, and evolution.. PLoS Genet.

[24] Dove SL, Hochschild A (2004). A bacterial two-hybrid system based on transcription activation.. Methods Mol Biol.

[25] Vallet-Gely I, Donovan KE, Fang R, Joung JK, Dove SL (2005). Repression of phase-variable cup gene expression by H-NS-like proteins in Pseudomonas aeruginosa.. Proc Natl Acad Sci U S A.

[26] Parsot C, Hamiaux C, Page AL (2003). The various and varying roles of specific chaperones in type III secretion systems.. Curr Opin Microbiol.

[27] Aizenman E, Engelberg-Kulka H, Glaser G (1996). An Escherichia coli chromosomal “addiction module” regulated by guanosine [corrected] 3′,5′-bispyrophosphate: a model for programmed bacterial cell death.. Proc Natl Acad Sci U S A.

[28] Hendrickson WA (1991). Determination of macromolecular structures from anomalous diffraction of synchrotron radiation.. Science.

[29] Kleanthous C, Walker D (2001). Immunity proteins: enzyme inhibitors that avoid the active site.. Trends Biochem Sci.

[30] Blower TR, Salmond GP, Luisi BF (2011). Balancing at survival's edge: the structure and adaptive benefits of prokaryotic toxin-antitoxin partners.. Curr Opin Struct Biol.

[31] Goodman AL, Kulasekara B, Rietsch A, Boyd D, Smith RS (2004). A signaling network reciprocally regulates genes associated with acute infection and chronic persistence in Pseudomonas aeruginosa.. Dev Cell.

[32] Wang J, Gossing M, Fang P, Zimmermann J, Li X (2011). Epsin N-terminal homology domains bind on opposite sides of two SNAREs.. Proc Natl Acad Sci U S A.

[33] Silverman JM, Austin LS, Hsu F, Hicks KG, Hood RD (2011). Separate inputs modulate phosphorylation-dependent and-independent type VI secretion activation.. Mol Microbiol.

[34] Hong W (2005). SNAREs and traffic.. Biochim Biophys Acta.

[35] Pedersen K, Christensen SK, Gerdes K (2002). Rapid induction and reversal of a bacteriostatic condition by controlled expression of toxins and antitoxins.. Mol Microbiol.

[36] Kelley LA, Sternberg MJ (2009). Protein structure prediction on the Web: a case study using the Phyre server.. Nat Protoc.

[37] Kamada K, Hanaoka F (2005). Conformational change in the catalytic site of the ribonuclease YoeB toxin by YefM antitoxin.. Mol Cell.

[38] Stover CK, Pham XQ, Erwin AL, Mizoguchi SD, Warrener P (2000). Complete genome sequence of Pseudomonas aeruginosa PA01, an opportunistic pathogen.. Nature.

[39] Kunkel TA (1985). Rapid and efficient site-specific mutagenesis without phenotypic selection.. Proc Natl Acad Sci U S A.

[40] Rietsch A, Vallet-Gely I, Dove SL, Mekalanos JJ (2005). ExsE, a secreted regulator of type III secretion genes in Pseudomonas aeruginosa.. Proc Natl Acad Sci U S A.

[41] Vance RE, Rietsch A, Mekalanos JJ (2005). Role of the type III secreted exoenzymes S, T, and Y in systemic spread of Pseudomonas aeruginosa PAO1 in vivo.. Infect Immun.

[42] Otwinowski Z, Minor W (1997). Processing of x-ray diffraction data collected in oscillation mode.. Methods Enzymol.

[43] Terwilliger TC, Berendzen J (1999). Automated MAD and MIR structure solution.. Acta Crystallogr D Biol Crystallogr.

[44] Murshudov GN, Vagin AA, Dodson EJ (1997). Refinement of macromolecular structures by the maximum-likelihood method.. Acta Crystallogr D Biol Crystallogr.

[45] Project CC (1994). The CCP4 Suite: Programs for Protein Crystallography.. Acta Crystallogr D Biol Crystallogr.

[46] McRee DE (1999). XtalView/Xfit–A versatile program for manipulating atomic coordinates and electron density.. J Struct Biol.

[47] Schrodinger LLC (2010).

[48] Potterton E, McNicholas S, Krissinel E, Cowtan K, Noble M (2002). The CCP4 molecular-graphics project.. Acta Crystallogr D Biol Crystallogr.

[49] Davis IW, Leaver-Fay A, Chen VB, Block JN, Kapral GJ (2007). MolProbity: all-atom contacts and structure validation for proteins and nucleic acids.. Nucleic Acids Res.

[50] Berman HM, Westbrook J, Feng Z, Gilliland G, Bhat TN (2000). The Protein Data Bank.. Nucleic Acids Res.

[51] Dove SL, Hochschild A (1998). Conversion of the omega subunit of Escherichia coli RNA polymerase into a transcriptional activator or an activation target.. Genes Dev.

[52] Whiteley M, Lee KM, Greenberg EP (1999). Identification of genes controlled by quorum sensing in Pseudomonas aeruginosa.. Proc Natl Acad Sci U S A.

