# Supplementary figures and images for: Structural Basis for Type VI Secretion Effector Recognition by a Cognate Immunity Protein
Source: PLoS Pathog. 2012 Apr 12;8(4):e1002613. doi: 10.1371/journal.ppat.1002613 (PMC3325213; doi:10.1371/journal.ppat.1002613)

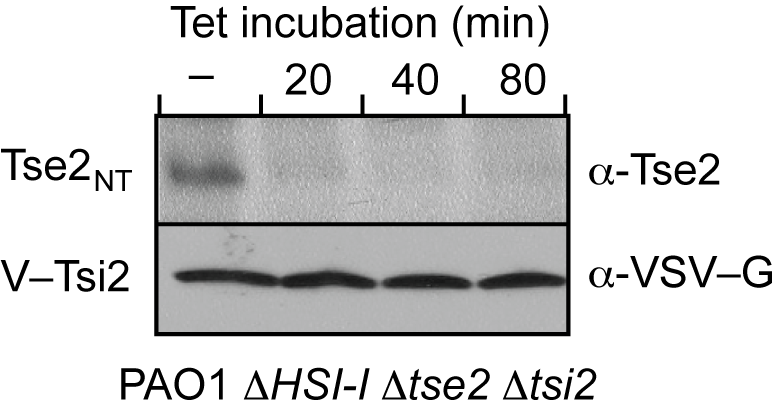

Supplement: Figure S1 — Epitope tags and export via the H1-T6SS do not account for the Tsi2 and Tse2 stability observed in Figures 2B and 3C , respectively. Analysis of Tse2NT and V–Tsi2 stability in P. aeruginosa ΔHSI-I Δtse2 Δtsi2 following the inhibition of protein synthesis by the addition of tetracycline (Tet). Top blot – the stability of untagged Tse2NT is similar to that observed for the C-terminally VSV–G fused protein (Fig. 3C). Bottom blot – N-terminally VSV–G fused Tsi2 (V–Tsi2) displays similar stability to the C-terminally-tagged protein (Fig. 2B). These experiments were conducted in an HSI-I deletion strain in order to rule out Tse2 secretion as a factor contributing to its depletion from cells. (TIF) [file ppat.1002613.s001.tif]

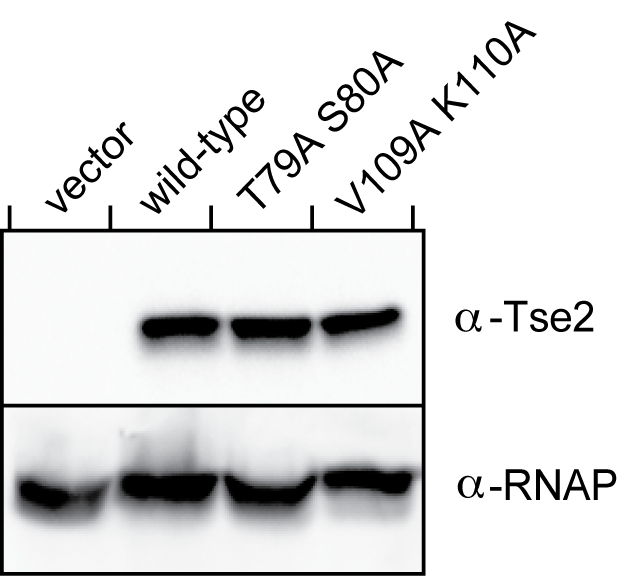

Supplement: Figure S2 — Non-toxic Tse2 alleles express to similar levels as the wild-type. Western blot analysis of the indicated tse2–V alleles expressed in P. aeruginosa Δtse2Δtsi2 grown on LB agar plates containing 30 µg/µl gentamycin and 0.5 mg/ml IPTG. RNA polymerase (RNAP) was included as a loading control. (TIF) [file ppat.1002613.s002.tif]

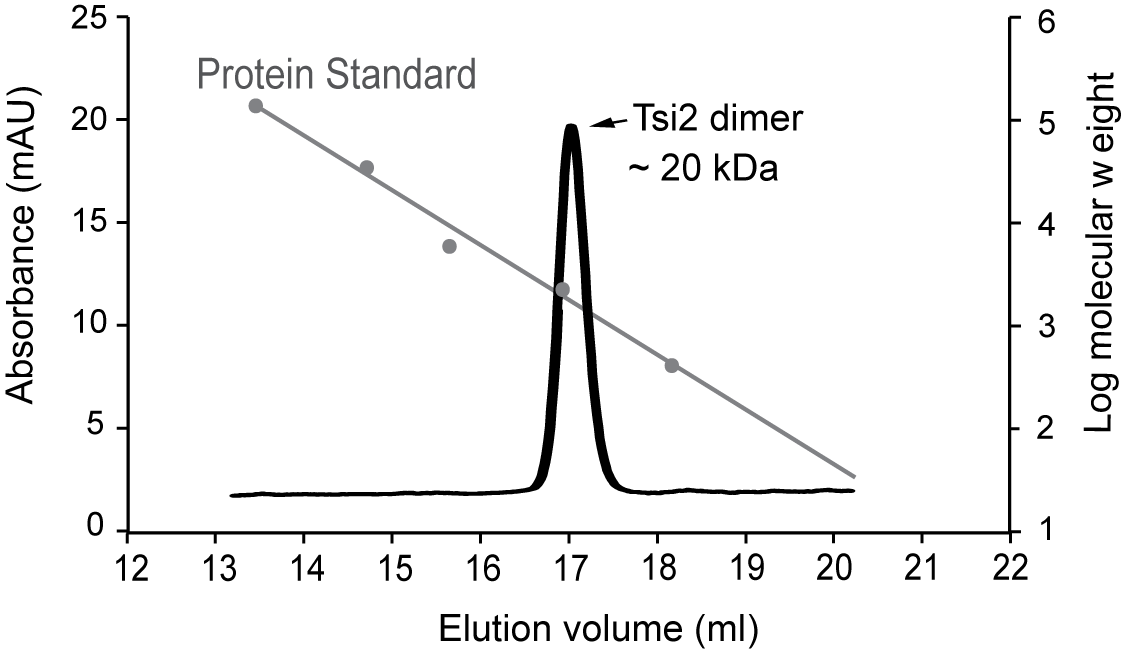

Supplement: Figure S3 — Analytical gel filtration elution profile of the Tsi2 dimer. Purified Tsi2–H6 was loaded onto a Superdex-200 10/300GL HR10/30 column (GE Health Care). Standard curves were calculated using linear regression analysis. (TIF) [file ppat.1002613.s003.tif]

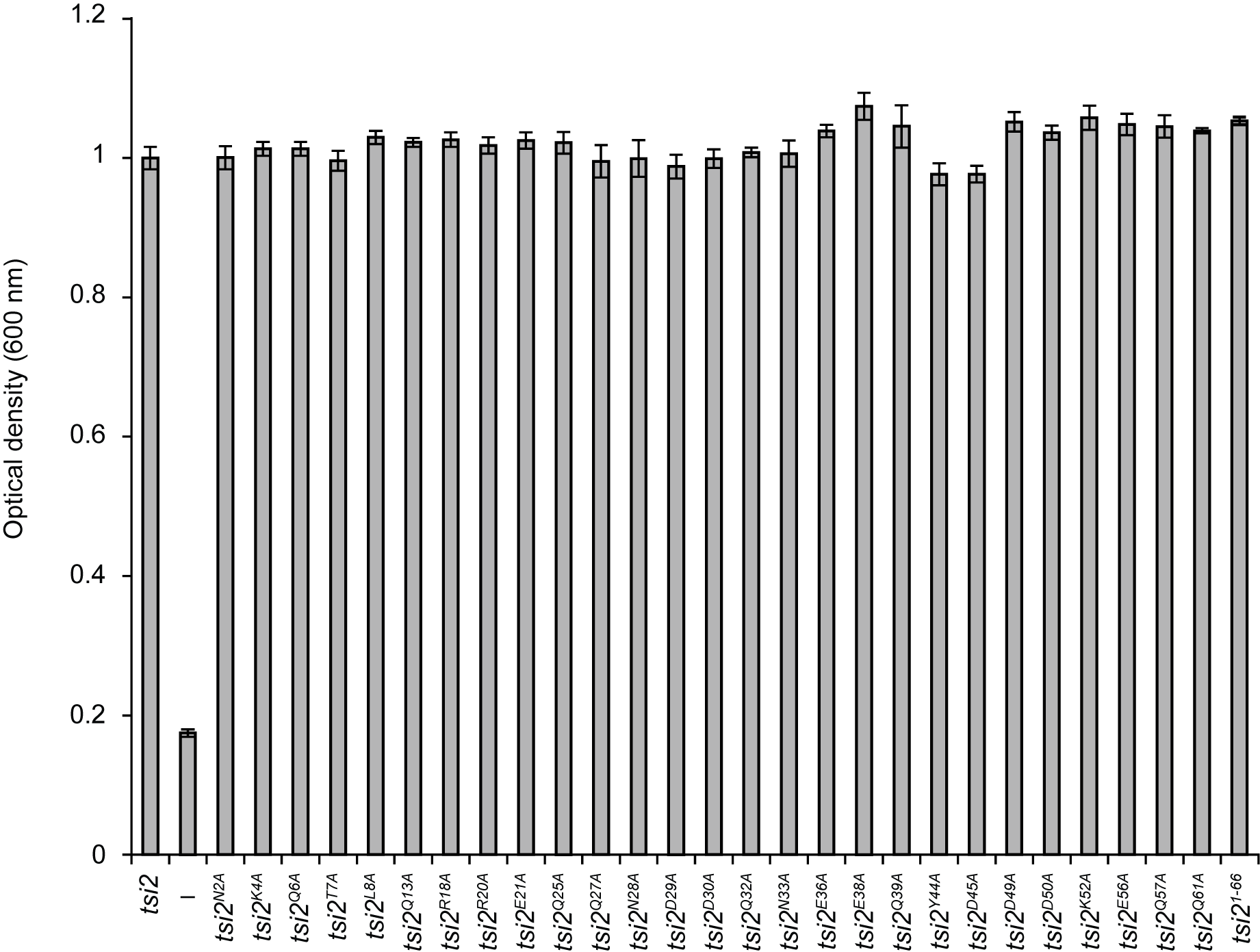

Supplement: Figure S4 — Tsi2 surface amino acid alanine substitutions do not affect its function. Growth of E. coli BL21 pLysS cells harboring pET29b+ co-expressing tse2 with the indicated tsi2–V alleles. Growth was measured in LB broth containing kanamycin using the automated BioScreen C Microbiology plate reader with agitation at 37°C. Bars represent growth normalized to tsi2 wild-type at six hours post-inoculation. Error bars represent standard deviation of three independent measurements. (TIF) [file ppat.1002613.s004.tif]

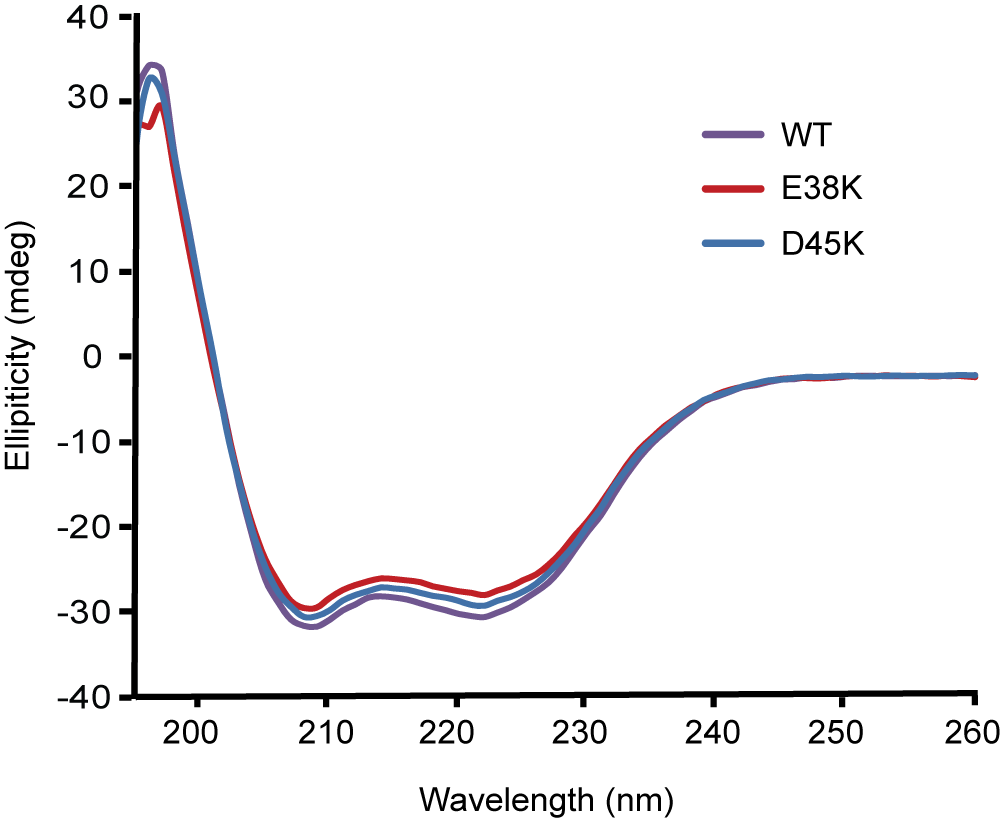

Supplement: Figure S5 — Substitution of Tsi2 acidic patch residues with lysine does not significantly alter structure. Far-UV CD spectrum of Tsi2–H6, Tsi2E38K–H6 and Tsi2D45K–H6 at 20°C. (TIF) [file ppat.1002613.s005.tif]

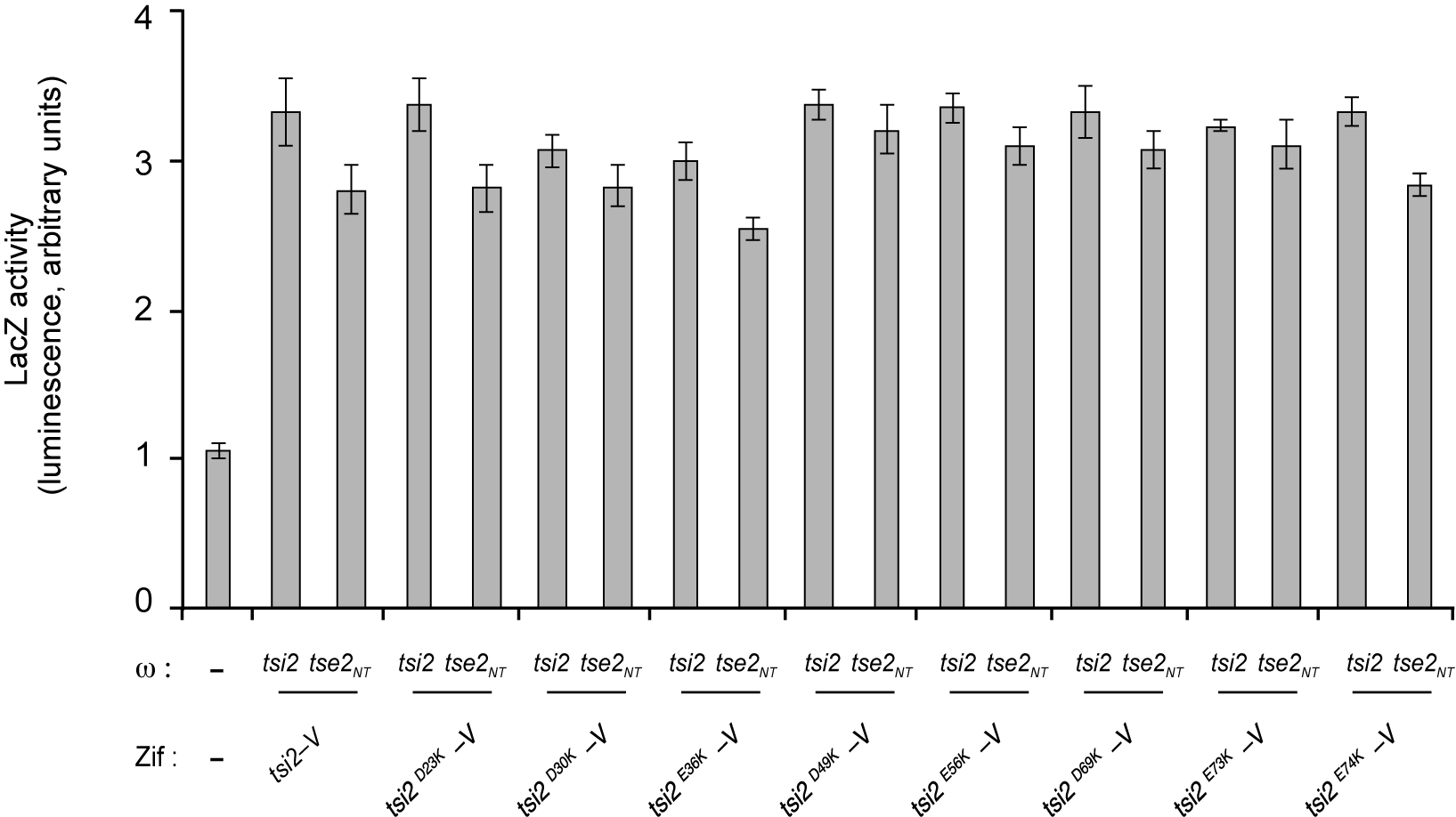

Supplement: Figure S6 — Tsi2 surface acidic residue lysine substitution mutants that do not impact homodimerization or interaction with Tse2. B2H analysis of Tsi2 acidic residue substitutions on homotypic (Tsi2) and heterotypic (Tse2) interactions. Genes fused in-frame to RNAP–ω and Zif are indicated. (TIF) [file ppat.1002613.s006.tif]

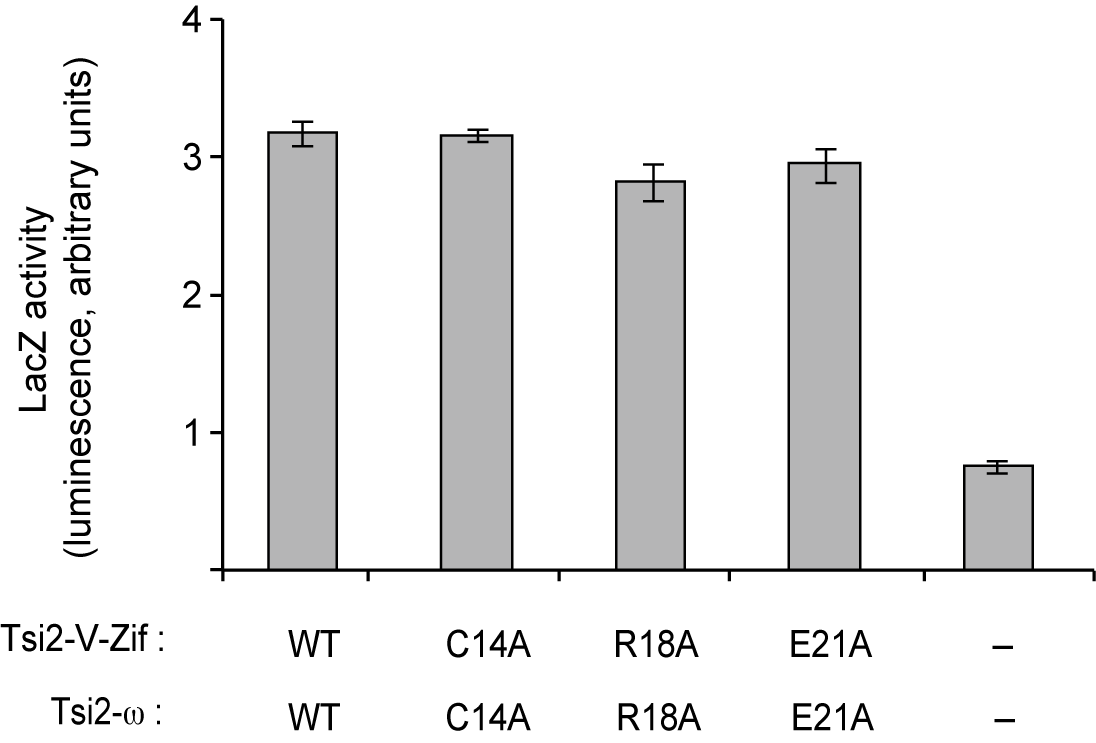

Supplement: Figure S7 — Nonconservative substitutions of Cys14 and polar dimer interface residues do not impact Tsi2 dimer formation. B2H analysis of Tsi2 dimer interface amino acid substitutions on Tsi2 dimerization. Genes fused in-frame to RNAP–ω and Zif are indicated. (TIF) [file ppat.1002613.s007.tif]
