# Supplementary material for: Structural Basis for Type VI Secretion Effector Recognition by a Cognate Immunity Protein
Source: PLoS Pathog. 2012 Apr 12;8(4):e1002613. doi: 10.1371/journal.ppat.1002613 (PMC3325213; doi:10.1371/journal.ppat.1002613)
Supplement: Table S1 — Tsi2 data collection, phasing and refinement statistics. (PDF) [file ppat.1002613.s008.pdf]

Table S1. Tsi2 data collection, phasing and refinement statistics

	Native	Se-Met Peak	Se-Met Remote
**Data collection**			
Space group	P1		
Cell dimensions			
a, b, c (Å)	27.8, 34.1, 37.7	27.8, 34.1, 37.7	27.8, 34.1, 37.7
α, β, γ (°)	110.5, 95.4, 95.8	110.5, 95.4, 95.8	110.5, 95.4, 95.8
Wavelength	0.9792	0.9793	0.9076
Resolution (Å)	50-0.99 (1.01-0.99)[a]	50-1.68 (1.71-1.68)	50-1.56 (1.59-1.56)
R_{merge}	0.035 (0.197)	0.032 (0.089)	0.032 (0.118)
I / σI	31.2 (3.7)	20.5 (8.1)	21.5 (5.9)
Completeness (%)	89.8 (45.0)	95.0 (83.5)	95.4 (87.0)
Redundancy	3.7 (2.4)	1.9 (1.5)	1.9 (1.5)
**Refinement**			
Resolution (Å)	34.88-1.00		
No. reflections	60525		
R_{work} / R_{free}	0.144/ 0.176		
No. atoms			
Protein	1363		
Water	188		
B-factors			
Protein	14.1		
Water	24.3		
R.m.s deviations			
Bond lengths (Å)	0.018		
Bond angles (°)	1.7		

[a] Values in parentheses are for highest-resolution shell.
